# Comparison of *Staphylococcus pettenkoferi* Isolated from Human Clinical Cases and Cat Carriers Regarding Antibiotic Susceptibility and Biofilm Production

**DOI:** 10.3390/ijms26051948

**Published:** 2025-02-24

**Authors:** Karolina Bierowiec, Ashley Delmar, Magdalena Karwańska, Magdalena Siedlecka, Aleksandra Kumala-Ćwikła, Marta Książczyk, Katarzyna Kapczyńska

**Affiliations:** 1 Department of Epizootiology and Clinic of Birds and Exotic Animals, Division of Infectious Diseases and Veterinary Administration, The Faculty of Veterinary Medicine in Wrocław, Wrocław University of Environmental and Life Sciences, 50-366 Wrocław, Poland; magdalena.karwanska@upwr.edu.pl (M.K.); magdalena.siedlecka@upwr.edu.pl (M.S.); aleksandra.kumala-cwikla@wroc.wiw.gov.pl (A.K.-Ć.); 2 EZA Student Science Club, Department of Epizootiology and Clinic of Birds and Exotic Animals, Division of Infectious Diseases and Veterinary Administration, The Faculty of Veterinary Medicine in Wrocław, Wrocław University of Environmental and Life Sciences, 50-366 Wrocław, Poland; 3 Department of Microbiology, Faculty of Biological Sciences, University of Wrocław, 51-148 Wrocław, Poland; marta.ksiazczyk@uwr.edu.pl; 4 Laboratory of Medical Microbiology, Hirszfeld Institute of Immunology and Experimental Therapy, Polish Academy of Sciences, 53-114 Wrocław, Poland; katarzyna.kapczynska@hirszfeld.pl

**Keywords:** *Staphylococcus*, multidrug resistance, One Health, *Galleria mellonella*

## Abstract

*Staphylococcus pettenkoferi (S. pettenkoferi)* is a rare opportunistic bacterium not commonly found in healthy individuals or animals*. S. pettenkoferi* has increasing clinical significance in both veterinary and human medicine due to its multidrug resistance and biofilm-forming ability. This study analyzed 12 isolates of *S. pettenkoferi* collected from humans and cats and identified them using matrix-assisted laser desorption/ionization time-of-flight mass spectrometry (MALDI-TOF MS) and *16S rRNA* and partial *rpo*B gene sequencing. All of the *S. pettenkoferi* were phenotypically resistant to penicillin, and almost all (except one human strain) were resistant to methicillin. Antibiotic susceptibility testing revealed a high prevalence of multidrug resistance in all human strains with frequent resistance to β-lactams, macrolides, and tetracyclines. A comparative analysis of human and feline isolates indicated the presence of shared resistance genes such as *blaZ*, *mecA*, and *ermA*. Biofilm production varied across isolates, with more potent biofilm formation abilities observed at elevated temperatures (39 °C) and time (48 h). These findings underscore the potential zoonotic risks of *S. pettenkoferi* and its role in managing multidrug-resistant infections.

## 1. Introduction

*Staphylococcus pettenkoferi (S. pettenkoferi)* is a rare staphylococcal species that has traditionally been described as a cause of bloodstream infections in humans [1,2,3,4]. Similar to other coagulase-negative staphylococci (CNS), *S. pettenkoferi* is a ubiquitous colonizer of skin and mucosal surfaces and plays a role as an opportunistic pathogen in immunocompromised patients [1,4,5]. *S. pettenkoferi* has also been isolated from wounds, ulcers, vaginal abscesses, and the frontal sinuses of humans [5,6,7]. Historically, most *S. pettenkoferi* isolates have been found among skin commensals and were considered clinically insignificant [6]. Although *S. pettenkoferi* appears to demonstrate similar pathogenicity and opportunistic potential as most other CNS, its role in human infections is still undetermined [6].

The first report of the presence of *S. pettenkoferi* in veterinary medicine was in 2013, when strains of the bacteria were isolated from a feeding dish and blanket found in a cat cage at a veterinary hospital [8]. While the bacteria are rarely isolated in animals, isolations of *S. pettenkoferi* from buccal samples of two rabbits, a cat, and a dog were reported [9]. In Poland, the prevalence of *S. pettenkoferi* in 520 healthy cats investigated as part of this study was 0.5% and bacterial isolates were found only in the conjunctival sacs [10]. According to the currently available literature, *S. pettenkoferi* was found to be present in the peritoneal fluid of a cat in India that was infected with peritonitis [11]. The strains isolated from the cat’s peritoneal fluid were multidrug-resistant, making them potentially dangerous to humans [8,9,11]. Previous reports concerning the antibiotic sensitivity of *S. pettenkoferi* strains pointed to the bacteria’s capability for resistance to multiple drug classes [8,11,12,13].

The increasing emergence of multidrug-resistant staphylococci poses a growing threat to both public health and veterinary medicine worldwide [6,8,11,13]. While much attention has been given to well-known pathogens such as *Staphylococcus aureus* in humans [14] and *Staphylococcus pseudintermedius* in cats and dogs (companion animals) [15]*,* less common species such as *S. pettenkoferi* are gaining significance due to their multidrug resistance profiles and potential similarity to other CNS in terms of their ability to cause opportunistic infections [6,11,13]. These bacteria have evolved mechanisms to resist a broad spectrum of antibiotics, complicating treatment and increasing the burden of infections in both human and veterinary medicine [11,13]. Horizontal gene transfer (HGT) and recombination are recognized as genetic drivers in CNS. They function as gene flow regulators and significant reservoirs of genetic diversity for the entire genus [16]. Similar to other CNS, *S. pettenkoferi* may be a source of antibiotic resistance and virulent genes [13]. Despite these growing concerns, the pathogenic mechanisms, antimicrobial resistance patterns, and biofilm-forming capabilities of *S. pettenkoferi* remain poorly understood, limiting the ability to address its clinical and public health implications more effectively. In this context, our study aimed to bridge gaps in *S. pettenkoferi* knowledge by evaluating *S. pettenkoferi* isolates from both human and feline hosts in Wrocław, Poland. Our investigation combined phenotypic and genotypic analyses to assess the antimicrobial resistance, biofilm-forming ability, and pathogenicity of *S. pettenkoferi* isolates. By evaluating these characteristics, our study sought to provide critical insights into the epidemiology, clinical significance, and potential zoonotic risk of this underexplored species. Our study also sought to contribute to the broader understanding of multidrug-resistant staphylococci from a One Health perspective.

## 2. Results

**Figure 1:**
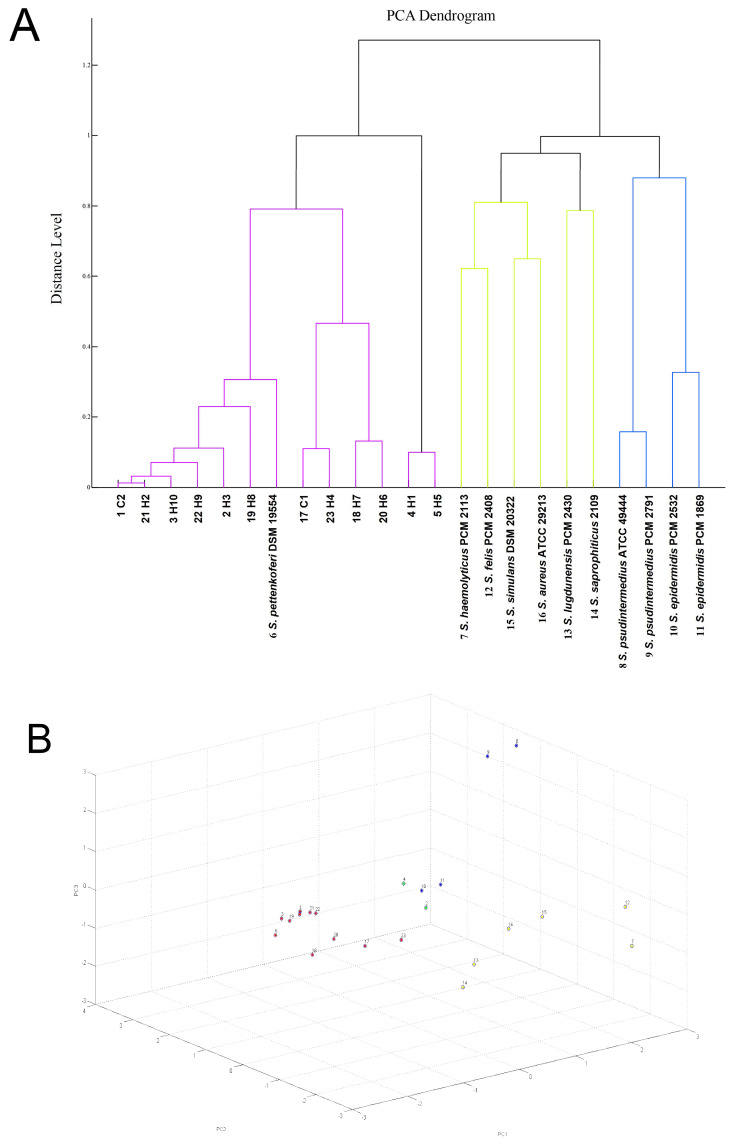
(**A**) Visual depiction of the closeness of individual spectra of *S. pettenkoferi* and chosen referential strains obtained with MALDI-TOF MS analysis. (**B**) PCA clustering of MALDI Biotyper spectra of *S. pettenkoferi* isolates and chosen referential strains: 1—C2, 2—H3, 3—H10, 4—H1, 5—H5, 6—*S. pettenkoferi* DSM 19554, 7—*S. haemolyticus* PCM 2113, 8—*S. pseudintermedius* ATCC 49444, 9—*S. pseudintermedius* PCM 2791, 10—*S. epidermidis* PCM 2532, 11—*S. epidermidis* PCM 1869, 12—*S. felis* PCM 2408, 13—*S. lugdunensis* PCM 2430, 14—*S. saprophyticus* 2109, 15—*S. simulans* DSM 20322, 16—*S. aureus* ATCC 29213, 17—C1, 18—H7, 19—H8, 20—H6, 21—H2, 22—H9, 23—H4.

All bacterial strains investigated as part of the study were identified as *S. pettenkoferi* using matrix-assisted laser desorption/ionization time-of-flight mass spectrometry (MALDI-TOF MS) with scores ≥ 2.0, the accepted species level identification score for *S. pettenkoferi.* A sequence analysis of the *16S rRNA* gene and partial *rpoB* gene sequencing were also used to identify the strains. Using the collected spectra and bacterial identification, a principle component analysis (PCA) dendrogram and cluster were obtained, allowing for a visual depiction of the closeness of individual spectra (listed on the *x*-axis) to one another (Figure 1). The resulting dendrogram showed that spectra obtained from the investigated *S. pettenkoferi* allowed the strains to be distinguished from other staphylococcal species for proper species identification. Figure S1 in the Supplementary Materials section depicts a comparison of the MALDI-TOF spectra for all investigated *S. pettenkoferi* strains. Identification of the strains was further supported by *16s rRNA* gene sequence analysis and by partial *rpoB* gene sequencing.

**Figure 2:**
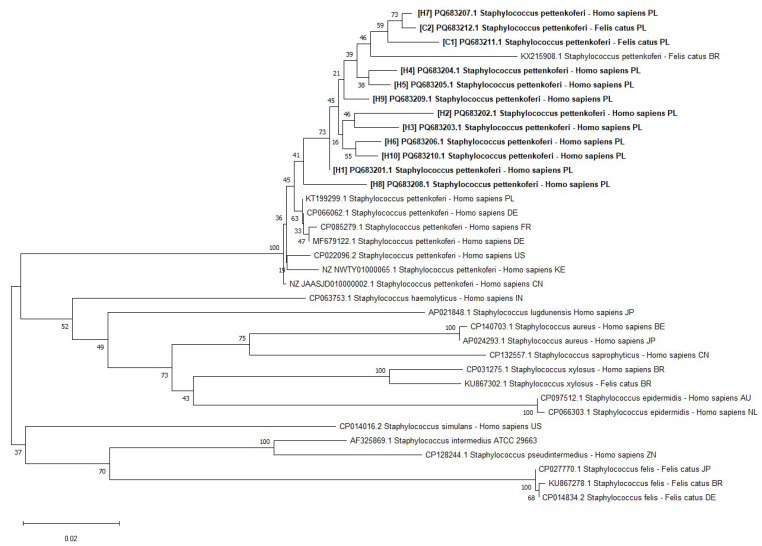
Phylogenetic tree based on partial *rpoB* gene sequencing of the genus *Staphylococcus*, including the sequences under investigation (C1, C2, H1–H10, indicated with bold font) and reference sequences from GenBank database. All sequences were obtained with MEGA 11. Genetic distance is indicated on the scale (the scale bar represents 2 nucleotide substitutions per 100 nucleotides).

The investigated *S. pettenkoferi* strains were also examined with partial *rpoB* gene sequencing. The strains were found to be highly similar to one another, with the investigated animal strains being genetically similar to the investigated human strains. The resulting dendrogram is presented in Figure 2.

All investigated *S. pettenkoferi* strains were shown to be phenotypically resistant to penicillin, and all except one human strain were found to be resistant to methicillin. PCR genetic resistance testing supported these results. All of the investigated strains harbored the *erm*A gene, while seven also showed resistance to erythromycin on the phenotypic level. Only the *S. pettenkoferi* strains isolated from cats were not found to be multidrug resistant on the phenotypic level, while all *S. pettenkoferi* strains under investigation were MDR (multidrug resistant) according to the presence of these genetic determinants of resistance. Detailed profiles of *S. pettenkoferi* phenotypic and genotypic resistance are presented in Table 1.

**Figure 3:**
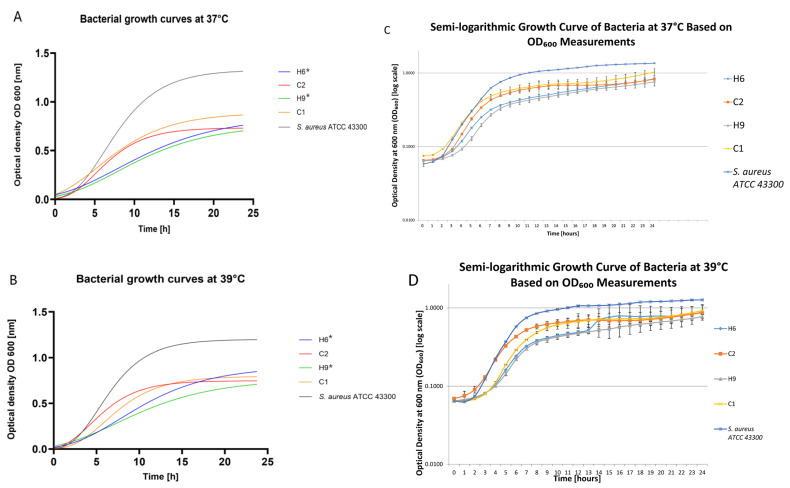
Growth curves of *S. pettenkoferi* of feline origin (C1 and C2) and human origin (H6 and H9) compared to *S. aureus* ATCC 43300 strain in (**A**) 37 °C and (**B**) in 39 °C; growth curves of bacterial strains (H6, C2, H9, C1, and *S. aureus* ATCC 43300) over a 24-h period in (**C**) 37 °C and (**D**) in 39 °C. Optical density (OD) at 600 nm was measured at hourly intervals. The data represent the mean OD values, with error bars indicating the standard deviation (SD), and the corresponding generation times (GT) ± SD. * a significant difference in growth compared to *S. aureus* ATCC 43300; *p* < 0.05.

Physiological feline body temperature oscillates around 39 °C; thus, our study investigated the growth of human and feline *S. pettenkoferi* strains at the standard 37 °C and at 39 °C for 48 h. The growth curves for selected *S. pettenkoferi* strains are presented in Figure 3 as well as by all strains in Figures S2–S5 in the Supplementary Materials. The growth of all *S. pettenkoferi* of human origin was significantly slower than that of *S. aureus* ATCC 43300 at 39 °C (*p* < 0.05), while there was no significant difference in the growth of feline strains either at 37 °C or 39 °C. The detailed data connected with the growth of strains under investigation are presented in the Supplementary Materials in Tables S4 and S5. The biofilm production properties in vitro were tested using a microtiter plate test (MPT) with crystal violet staining. Additionally, considering the slower growth of *S. pettenkoferi* in media, biofilm production was measured after 24 and 48 h. The *S. pettenkoferi* typically showed weak or medium biofilm production after 24 h of incubation, regardless of the temperature. However, biofilm production was shown to increase at higher temperatures and after prolonged incubation. Detailed data can be found in Table 2. The presence of the two biofilm-related genes, *ica*A and *bap*, was also investigated. None of the investigated *S. pettenkoferi* strains tested positive for these genes.

The minimum inhibitory concentration (MIC) and minimum bactericidal concentration (MBEC) of the chosen antimicrobials were also compared. Detailed data are shown in Table S2 in the Supplementary Materials. The general tendency shown was that much higher concentrations of antimicrobials were needed to demonstrate an inhibitory or bactericidal effect on biofilms than on the bacteria’s planktonic forms. Only in the case of clindamycin activity in five strains (H1, H3, H5, H6, H9) did we observe an unusual situation in which the results of the MIC analysis were higher or comparable to MBEC.

**Figure 4:**
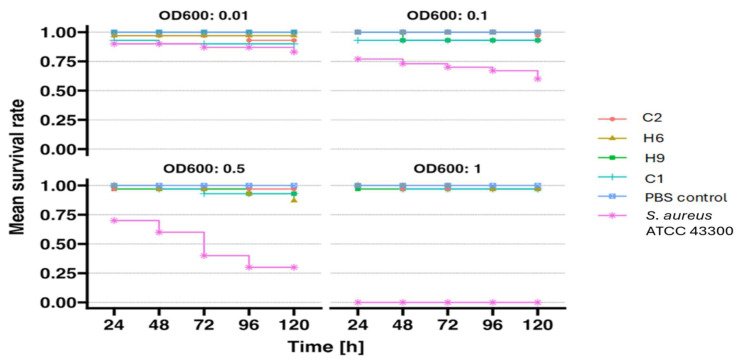
Survival curve of larvae [n = 10] following injection with the tested strains of *S. pettenkoferi*. The negative control consisted of larvae injected with sterile PBS buffer [PBS Control], while the positive control consisted of larvae injected with the *S. aureus* ATCC 43300 strain. Larval survival was monitored for 120 h.

None of the tested *S. pettenkoferi* strains exhibited pathogenicity towards the *G. mellonella* larva model, as shown in the larval survival curves in Figure 4 below. Each strain was administered at four different optical densities: OD1_600_ = 1, OD2_600_ = 0.5, OD3_600_ = 0.1, and OD4_600_ = 0.01. Regardless of the size of the inoculum, the larval mortality rate was negligible, ranging from 0% to a maximum of 13%, as detailed in Table S3A in the Supplementary Materials. All tests were performed with at least three independent replicates. As a positive control, *S. aureus* ATCC 43300 was tested, which showed significant pathogenicity in the larva model, resulting in 100% larval mortality following injection with the highest infectious dose of *S. aureus*. The obtained results were consistent with the calculated 50% lethal dose (LD_50_) for the tested *S. pettenkoferi* strains and the control strain *S. aureus* ATCC 43300 (Table S3B in the Supplementary Materials). The *S. pettenkoferi* strains exhibited a high LD_50_ value in *G. mellonella* larvae, ranging from 1.52 × 10^8^ to 2.42 × 10^14^, whereas the LD_50_ for the *S. aureus* ATCC 43300 strain was significantly lower, at 1.76 × 10^5^.

## 3. Discussion

*S. pettenkoferi* is a rare bacterium that is not commonly found in healthy individuals or companion animals [10]. CNS are often identified at the genus level only, as definitive species identification can be complex and costly [17]. This practice contributes to the difficulty in determining the true prevalence of specific CNS species, including *S. pettenkoferi*. The implementation of MALDI-TOF MS in routine bacteriological protocols can significantly improve the identification of CNS at the species level. Several studies demonstrated the reliability and accuracy of MALDI-TOF MS in identifying *S. pettenkoferi*, even in cases where traditional identification methods failed [18,19,20,21]. The results obtained in the current study support these observations.

Presently, the ability of humans to act as carriers of *S. pettenkoferi* has not been investigated. In the previous literature, *S. pettenkoferi* was described as the causative agent of bloodstream infections [2,3,7,22]; however, the source of the infections was not further investigated. The presence of *S. pettenkoferi* was traditionally categorized as a contaminant or as a colonizer of indwelling catheters and was regarded as clinically irrelevant [12,19]. It is unclear whether companion animals are the source of bacteria in their owners or if companion animals simply act as accidental hosts of *S. pettenkoferi*. Our previous studies showed that *S. pettenkoferi* is rarely isolated from companion animals [10,23]. Despite this, our comparison of *rpoB* sequencing showed high similarity between regional *S. pettenkoferi* strains of human and feline origin. Based on these results, the possibility of host affinity and interspecies transmission should be further investigated.

The pathogenicity potential of *S. pettenkoferi* is also unclear. In the current study, the presence of biofilm-encoding genes such as *bap* and *ica*A was not detected; however, biofilm-forming properties were proven using MPT. Gen *bap* (biofilm-associated protein) promotes both the initial adhesion of strains to abiotic surfaces and intercellular adhesion. While gen *ica*A is a part of *ica*ABCD, the operon encodes polysaccharide intercellular adhesin (PIA), the main component of biofilms [24]. Previous studies described *ica*A and *bap* as among the most frequently detected biofilm-encoding genes in CNS strains [24]. The *ica*ABCD operon is frequently found in invasive strains of CNS, making it a potential marker to distinguish CNS from less harmful strains [25]. Other reports, however, did not find a correlation between the operon *ica*ABCD and *bap* genes and biofilm-forming ability in phenotypic tests [24]. These biofilm-related genes were described previously in *S. pettenkoferi* strains [11,13,26]. Interestingly, previous investigations on clinical, animal, and environmental isolates reported no biofilm production [11,18]. Our study proves the biofilm-forming properties of all the *S. pettenkoferi* strains under investigation. The biofilm-forming ability was usually weak; nevertheless, the experiment showed that elongating the time of incubation and increasing the temperature improved biofilm production. In a study by Magnan et al. [13], 89.7% of isolates were able to produce biofilms and harbored a high content of biofilm-encoding genes. While *S. pettenkoferi* was associated with various infections, particularly in immunocompromised individuals, its specific features may predispose it to wound- and implant-related infections [5,13,26,27].

Due to their unique properties, such as the fact that their innate immune system displays many structural and functional similarities to the innate immune response of mammals, *G. mellonella* larvae serve as an ideal model for preliminary in vivo studie*s* [28]. The use of this organism complements commonly applied in vitro methods (e.g., cell lines or organoids) [28]. *G. mellonella* larvae were repeatedly and successfully used as models to study specific mechanisms of pathogenicity and virulence factors of *Staphylococcus* species [29,30,31]. This insect model provides a rapid and accessible tool for monitoring the involvement of sRNA and mRNA in *S. aureus* pathogenesis and can also be applied to other human bacterial pathogens [32]. *G. mellonella* can also be used for other purposes, including studying the interactions between *S. aureus* and larval immune response as well as how bacteria induce the expression of immune-related peptides [32]. In contrast to other alternative insect models such as *Caenorhabditis elegans* or *Drosophila melanogaster*, *G. mellonella* larvae can be infected at 37 °C, which is a significant advantage in studies of *Staphylococcus* virulence and other human and animal pathogens [33]. To date, the *G. mellonella* larvae model has not been used to assess the pathogenicity of *S. pettenkoferi* strains. In our study, we demonstrated the low pathogenicity of this bacterial species in *G. mellonella* larvae. We tested a broad inoculum ranging from 10^6^ to 10^9^ CFU/mL, obtaining the highest larval mortality (around 13%) after injecting a single strain of *S. pettenkoferi*. As a positive control, we used the *S. aureus* ATCC strain, which exhibited significant pathogenicity towards the larvae and caused 100% mortality. This proved the low pathogenicity of the *S. pettenkoferi* strains under investigation. Ahmad-Mansour et al. [26] assessed the pathogenicity of *S. pettenkoferi* strains in human serum, where the bacteria were able to survive. The team also conducted tests on human keratinocytes, mouse macrophages, and human macrophages and demonstrated the virulence of *S. pettenkoferi* in a zebrafish model, causing significant embryonic mortality in this species [26].

Research on the antibiotic resistance patterns of *S. pettenkoferi* is still relatively limited compared to more common staphylococcal bacteria such as *S. aureus*. As with other CNS, *S. pettenkoferi* has the potential to develop resistance to various classes of antibiotics. The current study also confirmed that almost all the investigated strains (11/12) showed phenotypic resistance to beta-lactams and harbored the *mec*A gene. The *erm*A gene was detected in all *the* strains of *S. pettenkoferi*, which was connected to phenotypic resistance to macrolides in more than half of the strains. Our team also observed *S. pettenkoferi’s* multidrug-resistant properties. Similar results were described in work by Park et al. [12], where almost all *S. pettenkoferi* were resistant to penicillin, oxacillin, ciprofloxacin, and erythromycin (5/6). In another study, by Magnan et al. [13], 41.4% of tested *S. pettenkoferi* were shown to be resistant to penicillin G, while only one strain carried the *mecA* gene and was methicillin-resistant. All isolates were susceptible to gentamicin. In a report on a clinical infection caused by *S. pettenkoferi* in a cat, the tested strain was resistant to 36 antimicrobial drugs. The strain was found to be sensitive only to gentamicin, fluoroquinolones (enrofloxacin, norfloxacin, and levofloxacin), carbapenems (imipenem and meropenem), and tetracycline [11].

This study highlights the importance of the accurate species-level identification of CNS, particularly *S. pettenkoferi*, using advanced techniques like MALDI-TOF MS. While previously overlooked or misidentified, *S. pettenkoferi* demonstrates potential pathogenicity, including biofilm-forming abilities and multidrug resistance. Our findings suggest a possible zoonotic transmission and emphasize the need for further investigation into this emerging pathogen’s epidemiology, virulence factors, and clinical significance to both humans and companion animals. The increasing prevalence of antibiotic resistance in *S. pettenkoferi* underscores the importance of appropriate antibiotic use and the development of new therapeutic strategies to effectively manage infections caused by this bacterium.

## 4. Materials and Methods

### 4.1. S. pettenkoferi Origin and Identification

In this study, *S. pettenkoferi* isolates from human and animal origin were investigated. Materials from human participants (n = 10; H1–10) obtained from the Microbiology Laboratory of the Wrocław Medical University Foundation in Wrocław, Poland, were investigated. All bacterial isolates were obtained from the blood of clinical patients between the years 2021 and 2022. The animal isolates (n = 2, C1 and C2) were obtained from the conjunctiva of clinically healthy feline patients at the Department of Epizootiology and Clinic of Birds and Exotic Animals, Faculty of Veterinary Medicine at Wrocław University of Environmental and Life Science in Wrocław, Poland, between the years 2013 and 2019. The sampling procedure and initial identification were performed as previously described in the literature [10].

The investigated bacterial strains were identified as *S. pettenkoferi* via the Biotyper platform using a Bruker matrix-assisted laser desorption/ionization time-of-flight (MALDI-TOF) ultrafleXtreme mass spectrometer. All bacterial strains were subjected to ethanol/formic acid extraction sample preparation procedures performed for MALDI Biotyper analysis. The MALDI Biotyper analysis was performed at least twice for all investigated strains and two technical spots were prepared for each extract. Overnight growing colonies were suspended in water and were adjusted with ethanol, thoroughly suspended, and centrifuged. After removing the supernatant, the pellet was mixed with an appropriate amount of 70% formic acid followed by the addition of acetonitrile and was then re-mixed. The sample was then recentrifuged and 1 µL of supernatant was pipetted onto a polished steel MALDI plate, dried, and covered with 1 µL of 10 mg/mL HCCA (alpha-cyano-4-hydroxycinnamic acid) matrix solution in acetonitrile:water:TFA (trifluoroacetic acid) solution (50:47.5:2.5). The cut-off for species identification was set at a score value of ≥2.000. The Biotyper platform allows for comparison of the protein profiles of unknown microbial isolates with reference to mass spectra in a database used to identify microorganisms. The result of identification with the highest score was used for further analysis. Principal component analysis (PCA) was applied to reduce the dataset’s dimensionality. The PCA analysis was based on the peak profiles obtained from MALDI-TOF mass spectrometry [34].

In addition to Biotyper identification, the isolates were also identified as *S. pettenkoferi* using BLAST analysis of the 16S rRNA PCR product [35]. The obtained sequences were identified by comparison with sequences available in the GenBank database using the BLAST search algorithm (http://blast.ncbi.nlm.nih.gov/Blast.cgi; accessed on 5 December 2024). The accession numbers of the isolated strains were PQ636928.1–PQ636939.1. Additionally, an analysis of the partial fragment of the *rpo*B gene was performed [36]. The *rpoB* sequences can be found in GenBank as PQ683201–PQ683212. A phylogenetic tree based on partial *rpoB* gene sequences was constructed using MEGA 11 software [37]. Distances were calculated according to the Kimura two-parameter model and clustering was based on the neighbor-joining method, along with bootstrapping for 1000 replicates.

### 4.2. Antibiotic Resistance and Virulence Factors of S. pettenkoferi

All *S. pettenkoferi* isolates were screened for antibiotic susceptibility using both phenotypic and genotypic methods. The phenotypic resistance was performed using the disk diffusion method [amoxicillin with clavulanic acid (30 μg), ampicillin (10 μg), cefoxitin (30 μg), chloramphenicol (30 μg), ciprofloxacin (5 μg), erythromycin (15 μg), gentamicin (10 μg), clindamycin (2 μg), fusidic acid (10 μg), linezolid (30 μg), mupirocin (200 μg), oxacillin (1 μg), penicillin G (10 IU), rifampicin (5 μg), sulfamethoxazole/trimethoprim (25 μg), tetracycline (30 μg), tigecycline (15 μg), tobramycin (10 μg) (Oxoid Ltd., Basingstoke, UK) and marbofloxacin (5 μg) (MASTDISCS^®^ *AST* (Mast Group Ltd., Liverpool, UK)] and MIC with the Sensititre™ EU Surveillance Staphylococcus EUST2 AST Plates (Thermo Fisher Scientific, Waltham, MA, USA). In the case of the MIC method, all solutions were allowed to reach room temperature prior to use. Three to five colonies were then selected from a fresh primary agar plate and suspended in sterile 0.9% NaCl, adjusted to 0.5 McFarland. Next, 100 µL of the suspension was transferred to a tube with 11 mL of Mueller–Hinton broth and was vortexed. Finally, 50 µL of the broth suspension was transferred to each well of the plate. Additionally, methicillin and teicoplanin resistances were detected using the broth microdilution method. The examined strains were inoculated in Mueller–Hinton broth (Oxoid, Basingstoke, UK) in a dilution series of oxacillin (16, 8, 4, 2, 1, 0.5, 0.25, 0.125, 0.0625, and 0.03125 µg/mL) and teicoplanin (128, 64, 32, 16, 8, 4, 2, 1, 0.5 µg/mL) (TOKU-E, Gent, Belgium) in water [38]. The inoculum was prepared according to the standard broth microdilution procedure described in the Clinical and Laboratory Standards Institute (CLSI) Performance Standards for Antimicrobial Disk and Dilution Susceptibility Tests for Bacteria Isolated From Animals, supplement VET01S [35]. Methicillin-resistant strains were detected using the CLSI criteria for CNS species for human and feline isolates, respectively [39,40]. *S. aureus* ATCC 29213 was used as a negative control, and *S. aureus* ATCC 43300 (*mec*A-positive) was used as a positive control. The double disk diffusion test (D-test) was performed on all isolates to detect inducible clindamycin resistance [41].

Antibiotic-resistant genotypes were identified using PCR analysis. The presence of genes involved in resistance to penicillin, aminoglycosides, β-lactamase, glycopeptides, macrolide-lincosamide-streptogramin, tetracyclines, fusidic acid, chloramphenicols, and mupirocin was determined using PCR. The detailed methods involved with the genetic determination of resistance and virulence factors are described in the Supplementary Materials (Table S1). *S. pettenkoferi* isolates that were found to be resistant to three or more classes of antimicrobial agents were interpreted as being MDR.

### 4.3. Growth Curves’ Evaluation and Biofilm Formation

The growth profiles of all *S. pettenkoferi* strains and reference strains were investigated using brain infusion bullion (BHI) (Oxoid, Basingstoke, UK). The overnight suspension of each strain in 5 mL of BHI was diluted to an optical density of around 0.1 at 600 nm (Eppendorf BioPhotometer 6131, Hamburg, Germany). Then, 500 µL of each bacterial suspension was added to three wells of a 48-well plate, incubated, and automatically shaken for 48 h at 37 °C and 39 °C. Each well’s absorbance at 600 nm was determined using an automatic absorbance reader (Spark Tecan, Männedorf, Switzerland). Growth curves were determined using GraphPad Prism 10.4.1 software (San Diego, CA, USA), as it was previously described by Magnan et al. (2022) [13]. To estimate growth kinetics, the per-capita growth rate was computed from OD readings using the calc_deriv function. The doubling time was then calculated using the doubling_time function. Data were analyzed in R 4.4.1 using the gcplyr 1.11.0 package [42]. The differences in the growth of *S. pettenkoferi* strains under investigation at 37 °C and 39 °C and comparison to *S. aureus* ATCC 43300 were investigated using the Mann–Whitney test with statistical significance at *p* < 0.5.

The presence of the two biofilm-related genes*, ica*A and *bap*, was determined by duplex polymerase chain reaction (PCR), as it was previously described in the literature [43], with the positive controls *S. epidermidis* PCM 2532 and *S. epidermidis* AIR08630, respectively.

All strains were tested for slime production using the static MTP test with crystal violet staining in a 96-well plate. After an overnight culture of all strains under investigation in 5 mL of BHI (Oxoid, Basingstoke, UK), the culture was diluted 1:100 in BHI and 200 μL of the suspensions was added into three wells in four plates each and incubated for 24 and 48 h at 37 °C and 39 °C. After incubation, the plates were washed twice with 200 μL of sterile phosphate-buffered saline. Next, the plate was allowed to air-dry for 2 h before being stained with 200 μL of 0.1% crystal violet for 10 min. After the subsequent washing (as described above), the staining bound to the bacteria was dissolved by adding 250 μL of 95% ethanol. The absorbance values were determined at 570 nm on a plate reader (Infinite F50, Tecan, Männedorf, Switzerland) after 24 and 48 h of incubation at 37 °C and 39 °C. Interpretation was performed as previously described by Płoneczka et al. [43]. The MTP procedure was carried out three times, in triplicate for each strain under investigation. The Minimal Biofilm Eradication Concentration was also calculated for selected antimicrobials. Serial dilutions of the antimicrobials were used in the following concentrations: clindamycin (TOKU-E, Gent, Belgium) at 16, 8, 4, 2, 1, 0.5, 0.25, 0.125, and 0.0625 µg/mL; vancomycin (TOKU-E, Gent, Belgium) and teicoplanin TOKU-E, Gent, Belgium) at 128, 64, 32, 16, 8, 4, 2, 1, and 0.5 µg/mL; and erythromycin and tetracycline (TOKU-E, Gent, Belgium) at 32, 16, 8, 4, 2, 1, 0.5, and 0.25 µg/mL. The tests were conducted and interpreted as previously described by Dudek et al. [44].

### 4.4. Pathogenicity Tests on Larvae Model

#### 4.4.1. Preparation of Bacterial Suspension

Overnight cultures of the tested *S. pettenkoferi* strains [n = 4] were centrifuged at 3500 RPM. The resulting bacterial pellets were washed three times with sterile PBS buffer and then resuspended in PBS buffer to achieve an optical density of (1) OD_600_ = 1, (2) OD_600_ = 0.5, (3) OD_600_ = 0.1, and (4) OD_600_ = 0.01. The CFU/mL value for the prepared bacterial suspensions was determined using the culture method.

#### 4.4.2. Larval Selection and Injection

The experiment used larvae from a self-maintained culture at the Department of Epizootiology and Clinic of Birds and Exotic Animals at Wrocław University of Environmental and Life Sciences. Larvae weighing 300 ± 30 mg were selected and placed in groups of n = 10 on sterile Petri dishes. Each larva was injected with 10 μL of bacterial suspension directly into the hemocoel using a Hamilton Bonaduz 100 μL microliter syringe. Negative control: larvae [n = 10] injected with 10 μL sterile PBS buffer. Positive control: larvae [n = 10] injected with 10 μL of suspension of *Staphylococcus aureus* MRSA strain ATCC 43300. The suspension of the reference strain was prepared as described above for *S. pettenkoferi*. The experiment was repeated at least three times. Larval survival was monitored over 120 h at 37 °C after inoculation, counting the numbers of live and dead larvae.

#### 4.4.3. Analysis of the Results

The obtained data were analyzed statistically using GraphPad Prism software with a Kaplan–Meier algorithm. Survival curves were plotted based on the results. From the survival curves, the 50% lethal dose (LD_50_) for each bacterial strain was calculated. The LD_50_ represents the CFU/mL value of a given bacterial strain at which 50% of the larval population died. Calculations were performed using the method modified by Reed and Muench [45].

The percentage of larval mortality was calculated by determining the ratio of the number of dead larvae after 120 h of observation to the total number of larvae [n = 10] used for injection in a single experiment. The calculations were based on the average values for each bacterial strain and a specific CFU/mL concentration.

## Figures and Tables

**Table 1:** The phenotypic and genotypic antimicrobial profiles of all *S. pettenkoferi* strains under investigation were determined using the MIC, disk diffusion method, and PCR reactions.

ID	Phenotypic Antimicrobial Profile	Genotypic Antimicrobial Profile
C1	P, OX, C	blaZ, mecA, tet(L), ermA, vanA, aph3-IIIa
C2	P, OX	blaZ, mecA, ermA, vanA
H1	P, OX, AMP, AMC, CIP, MAR, E, CL, SMX	mecA, tet(L), ermA
H2	P, OX	blaZ, mecA, ermA, aph3-IIIa
H3	P, OX, AMP, AMC, CIP, MAR, E, CL, SMX	blaZ, mecA, tet(L), ter(M), ermA, aph3-IIIa
H4	P, OX, AMC, TET	blaZ, mecA, tet(M), ermA, fusB, aph3-IIIa
H5	P, OX, AMP, AMC, CIP, MAR, E, CL, SMX, TMP, FD	blaZ, mecA, tet(L), tet(M), ermA, mupA, fusB, aph3-IIIa
H6	P, OX, AMP, AMC, CIP, MAR, E, CL, SMX, TMP, FD, MUP	blaZ, mecA, tet(L), ermA, mupA, fusB, aph3-IIIa
H7	P, CIP, E, CL, SMX	blaZ, mecA, tet(L), ermA, vanA
H8	P, OX, TET, SMX	mecA, tet(L), ermA
H9	P, OX, AMP, AMC, CIP, MAR, E, CL, SMX, TMP, FD, MUP	blaZ, mecA, tet(L), ermA
H10	P, OX, AMC, E, CL, SMX	blaZ, mecA, tet(L), ermA

C1–C2: cats’ *S. pettenkoferi* strains; H1–H10: humans’ *S. pettenkoferi* strains; P = penicillin; OX = oxacillin, AMP—ampicillin; AMC = amoxicillin-clavulanate; CIP = ciprofloxacin; MAR = marbofloxacin; E = erythromycin; CL = clindamycin; TET = tetracycline; SMX = sulfamethoxazole; TMP = trimethoprim; FD = fusidic acid; MUP = mupirocin; C = chloramphenicol.

**Table 2:** The results of slime production by microtiter plate (MTP) test with crystal violet staining of *S. pettenkoferi* strains of human and feline origin after 24 and 48 h of incubation at 37 °C and 39 °C.

ID	Biofilm Production at 37 °C		Biofilm Production at 39 °C	
	After 24 h	After 48 h	After 24 h	After 48 h
C1	weak	medium	medium	strong
C2	weak	strong	medium	strong
H1	weak	medium	weak	strong
H2	weak	weak	weak	medium
H3	weak	weak	weak	medium
H4	weak	weak	weak	strong
H5	weak	weak	weak	medium
H6	weak	medium	weak	strong
H7	weak	weak	weak	medium
H8	weak	medium	weak	medium
H9	not present	medium	weak	strong
H10	weak	medium	weak	strong
*S. pettenkoferi* DSM 19554	weak	medium	weak	medium
*S. aureus* ATCC 43300	medium	strong	medium	strong
*S. aureus* ATCC 6532	strong	strong	strong	strong
*S. aureus* ATCC 11632	weak	medium	medium	strong
*S. pseudintermedius* PCM 2791	strong	strong	strong	strong
*S. pseudintermedius* ATCC 49444	strong	strong	strong	strong

According to microtiter plate assay results; weak biofilm formers, 0.252 ≤ A570 < 0.504; medium-positive biofilm formers, 0.504  ≤  A570  <  1.008; strong biofilm formers, A570  ≥  1.008.

## Data Availability

All data are available in the article and Supplementary Materials.

## Supplementary Materials

The supporting information can be downloaded at https://www.mdpi.com/article/10.3390/ijms26051948/s1. References [46,47,48,49,50,51,52,53,54,55,56,57,58] are cited in the Supplementary Materials.
