# Supplementary material for: Comparison of Staphylococcus pettenkoferi Isolated from Human Clinical Cases and Cat Carriers Regarding Antibiotic Susceptibility and Biofilm Production
Source: Int J Mol Sci. 2025 Feb 24;26(5):1948. doi: 10.3390/ijms26051948 (PMC11900592; doi:10.3390/ijms26051948)
Supplement: Supplementary file 1 [file ijms-26-01948-s001.zip › ijms-3417408-supplementary.pdf]

Table S1. Primers used in this study.

Target gene	Primer sequence (5'-3')	Amplicon size (bp)	Annealing temperature (°C)	Reference
**Primers used for classical PCR[A]**				
aac(6')-Ie/aph(2")	F: CCAAGAGCAATAAGGGCATACC<br>R: CACACTATCATAACCACTACCG	222	45	[1]
aac(6')Ie/aph(2")Ia	F: GAGCAATAAGGGCATACCAAAAATC<br>R: CCGTGCATTTGTCTTAAAAAACTGG	480	54	[2]
ant(4')-Ia	F: AATCGGTAGAAGCCCAA<br>R: GCACCTGCCATTGCTA	135	47	[1]
aph(3')-IIIa	F: CTGATCGAAAAATACCGCTGC<br>R: TCATACTCTTCCGAGCAAAGG	269	51	[1]
cat	F: GCGAACGAAAAACAATTGCA<br>R: TGAAGCTGTAAGGCAACTGG	748	56	[3]
cfr	F: TGAAGTATAAAGCAGGTTGGGAGTCA<br>R: ACCATATAATTGACCACAAGCAGC	746	56	[3]
ermB	F: GAAAAGGTACTCAACCAAATA<br>R: AGTAACGGTACTTAAATTGTTTAC	639	52	[2]
ermC	F: TCAAAACATAATATAGATAAA<br>R: GCTAATATTGTTTAAATCGTCAAT	642	52	[2]
fexA	F: GTACTTGTAGGTGCAATTACGGCTGA<br>R: CGCATCTGAGTAGGACATAGCGTC	1272	56	[3]
fusB	F: CCGTCAAAGTTATTCAATCG<br>R: ACAATGAATGCTATCTCGACA	492	50	[2]
mecC	F: TCACCAGGTTCAACyCAAAA<br>R: CCTGAATCwGCTAATAATATTTC	356	55	[2]
mupA	F: TATATTATGCGATGGAAGGTTGG<br>R: AATAAAATCAGCTGGAAAGTGTTG	457	53	[2]
vanA	F: GGGAAAACGACAATTGC<br>R: GTACAATGCGGCCGTTA	732	54	[2]
vanB	F: ATGGGAAGCCGATAGTC<br>R: GATTTCGTTCCTCGACC	635	54	[2]
vatA	F: TGGTCCCGGAACAACATTTAT<br>R: TCCACCGACAATAGAATAGGG	268	55	[4]
vatB	F: GCTGCGAATTCAGTTGTTACA<br>R: CTGACCAATCCCACCATTTTA	136	55	[4]
vatC	F: AAGGCCCCAATCCAGAAGAA<br>R: TCAACGTTCTTTGTCACAACC	467	55	[4]

tufA	F: GGCCGTGTTGAACGTGGTCAAATCA<br>R: TIACCATTTCAGTACCTTCTGGTAA	370	50	[5]
clf	F: GCAAAATCCAGCACAACAGGAAACGA<br>R: CTTGATCTCCAGCCATAATTGGTGG	638	55	[6]
sea	F: CAGCATACTATATTGTTTAAAGGC<br>R: CCTCTGAACCTTCCCATC	400	51	[7]
seb	F: GTATGGTGGTGTAACTGAGCA<br>R: TCAATCTTCACATCTTTAGAATCA	351	51	[7]
sec	F: CTCAAGAACTAGACATAAAAGCTAGG<br>R: TCAAAATCGGATTAACATTATCC	271	51	[8]
sed	F: CTAGTTTGGTAATATCTCCTTTAAACG<br>R: TTAATGCTATATCTTATAGGGTAAACATC	319	51	[8]
seh	F: CAACTGCTGATTTAGCTCAG<br>R: GTCGAATGAGTAATCTCTAGG	360	51	[9]
**Primers used for qRT-PCR[B]**				
blaZ	F: GCTTTAAAAGAACTTATTGAGGCTTCA<br>R: CCACCGATyTCkTTTATAATTT<br>probe: 6-FAM AGTGATAATACAGCAAACAA BHQ-1	80	55	[10]
ermA	F: TCAGTTACTGCTATAGAAATTGATGGAG<br>R: ATACAGAGTCTACACTTGGCTTAGG<br>probe: 6-FAM AGTGACTAAAGAAGCGGTAAACCCCTCTGA BHQ-1	358	55	[11]
mecA	F: AACCACCCAATTTGTCTGCC<br>R: TGATGGTATGCAACAAGTCGTAAA<br>probe: Hex CCTTGTTTCATTTTGAGTTCTGCAGTACCGG BHQ-1	135	55	[12]
tetK	F: CTAACCCTTCACCTAAAGCTAC<br>R: CTGTCTTGGTTCATTGATTGC<br>probe: 6-FAM AGCTACAACCACCATAATCAGTGAAGGGAA BHQ-1	197	59	This study
tetL	F: GGTTTTGAACGTCTCATTACCTGAT<br>R: CCAATGGAAAAGGTTAACATAAAGG<br>probe: Hex CCACCTGCGAGTACAAACTGGGTGAAC BHQ-1	63	55	[13]
tetM	F: GGTTTCTCTTGGATACTTAAATCAATCr<br>R: CCAACCATAyAATCCTTGTTCrC<br>probe: Hex ATGCAGTTATGGArGGGATACGCTATGG BHQ-1	88	55	[13]
tetO	F: AAGAAAACAGGAGATTCCAAAACG<br>R: ATAGGAAGTATAAGTAGGTAAGAC<br>probe: 6-FAM ACGTTATTTCCCGTTTATCACGG BHQ-1	76	55	[13]

[A]The classical PCR amplification conditions were an initial denaturation at 95°C for 5 min; 35 cycles of 95°C - 30 s, X°C - 30 s and 72°C - 1 min; followed by a final extension step at 72°C for 10 min, were X means annealing temperature.
[B]The qRT-PCR amplification conditions were an initial denaturation at 95°C for 3 min; 40 cycles of 95°C - 15 s, X°C - 1 min, were X means annealing temperature.

References

1. Mahdiyoun, S.M.; Kazemian, H.; Ahanjan, M.; Houri, H.; Goudarzi, M. Frequency of Aminoglycoside-Resistance Genes in Methicillin-Resistant *Staphylococcus aureus* (MRSA) Isolates from Hospitalized Patients. *Jundishapur J Microbiol*. **2016,** *9(8)*:e35052. doi:10.5812/jjm.35052

2. Bierowiec, K.; Płoneczka-Janeczko, K.; Rypuła, K. Diversity of antimicrobial-resistant pheno- and genotypes of *Staphylococcus aureus* from clinically healthy cats kept in city households. *Berliner und Munchener Tierarztliche Wochenschrift*. **2017,** *130*:50–57. doi:10.2376/0005-9366-16043

3. Udo, E.E.; Boswihi, S.S.; Mathew, B.; Noronha, B.; Verghese, T. Resurgence of Chloramphenicol Resistance in Methicillin-Resistant *Staphylococcus aureus* Due to the Acquisition of a Variant Florfenicol Exporter (*fexAv*)-Mediated Chloramphenicol Resistance in Kuwait Hospitals. *Antibiotics (Basel)*. **2021,** *10(10)*:1250. doi:10.3390/antibiotics10101250

4. Strommenger, B.; Kettlitz, C.; Werner, G.; Witte, W. Multiplex PCR assay for simultaneous detection of nine clinically relevant antibiotic resistance genes in *Staphylococcus aureus*. *J Clin Microbiol*. **2003,** *41(9)*:4089-4094. doi:10.1128/JCM.41.9.4089-4094.2003

5. Martineau, F.; Picard, F.J.; Ke, D.; Paradis, S.; Roy, P.H.; Ouellette, M.; Bergeron, M.G. Development of a PCR assay for identification of staphylococci at genus and species levels. *J Clin Microbiol*. **2001,** *39(7)*:2541-2547. doi:10.1128/JCM.39.7.2541-2547.2001

6. Mason, W.J.; Blevins, J.S.; Beenken, K.; Wibowo, N.; Ojha, N.; Smeltzer, MS. Multiplex PCR protocol for the diagnosis of staphylococcal infection. *J Clin Microbiol*. **2001,** *39(9)*:3332-3338. doi:10.1128/JCM.39.9.3332-3338.2001

7. Park, J.Y.; Fox, L.K.; Seo, K.S.; McGuire, M.A.; Park, Y.H.; Rurangirwa, F.R.; Sischo, W.M.; Bohach, G.A. Detection of classical and newly described staphylococcal superantigen genes in coagulase-negative staphylococci isolated from bovine intramammary infections. *Vet Microbiol*. **2011,** *147(1-2)*:149-154. doi:10.1016/j.vetmic.2010.06.021

8. Becker, K.; Roth, R.; Peters, G. Rapid and specific detection of toxigenic *Staphylococcus aureus*: use of two multiplex PCR enzyme immunoassays for amplification and hybridization of staphylococcal enterotoxin genes, exfoliative toxin genes, and toxic shock syndrome toxin 1 gene. *J Clin Microbiol*. **1998,** *36(9)*:2548-2553. doi:10.1128/JCM.36.9.2548-2553.1998

9. Monday, S.R.; Bohach, G.A. Use of multiplex PCR to detect classical and newly described pyrogenic toxin genes in staphylococcal isolates. *J Clin Microbiol*. **1999,** *37(10)*:3411-3414. doi:10.1128/JCM.37.10.3411-3414.1999

10. Pereira, L.A.; Harnett, G.B.; Hodge, M.M.; Cattell, J.A.; Speers, D.J. Real-time PCR assay for detection of blaZ genes in *Staphylococcus aureus* clinical isolates. *J Clin Microbiol*. **2014,** *52(4)*:1259-1261. doi:10.1128/JCM.03413-13

11. Jung, J.H.; Yoon, E.J.; Choi, E.C.; Choi, S.S. Development of TaqMan probe-based real-time PCR method for erm(A),erm(B), and erm(C), rapid detection of macrolide-lincosamide-streptogramin B resistance genes, from clinical isolates. *J Microbiol Biotechnol*. **2009,** *19(11)*:1464-1469. doi:10.4014/jmb.0902.062

12. Kelley, K.; Cosman, A.; Belgrader, P.; Chapman, B.; Sullivan, DC. Detection of methicillin-resistant *Staphylococcus aureus* by a duplex droplet digital PCR assay. *J Clin Microbiol*. **2013,** *51(7)*:2033-2039. doi:10.1128/JCM.00196-13

13. Peak, N.; Knapp, C.W.; Yang, R.K.; Hanfelt, M.M.; Smith, M.S.; Aga, D.S.; Graham, D.W. Abundance of six tetracycline resistance genes in wastewater lagoons at cattle feedlots with different antibiotic use strategies. *Environ Microbiol*. **2007,** *9(1)*:143-151. doi:10.1111/j.1462-2920.2006.01123.x

Table S2. Comparison of Minimum Inhibitory Concentration (MIC) and Minimum Biofilm Eradication Concentration (MBEC) towards oxacillin, teicoplanin, erythromycin, clindamycin, vancomycin and tetracycline, and *S. pettenkoferi* strains of human and cat origin.

	**teicoplanin** μg/mL		**erythromycin** μg/mL		**clindamycin** μg/mL		**vancomycin** μg/mL		**tetracycline** μg/mL	
**ID**	**MIC**	**MBEC**	**MIC**	**MBEC**	**MIC**	**MBEC**	**MIC**	**MBEC**	**MIC**	**MBEC**
C1	2	128	0.5	32	0.125	4	4	16	<0.5	32
C2	2	>128	<0.25	>32	<0.12	16	2	>128	<0.5	>32
H1	4	128	≥16	>32	≥8	16	<1	>16	<0,5	>32
H2	2	16	<0.25	>32	<0.12	>16	<1	128	<0.5	16
H3	2	32	≥16	32	≥8	8	2	32	<0.5	32
H4	2	32	<0.25	16	<0.12	8	<1	4	0.125	32
H5	2	>128	≥16	>32	≥8	2	2	>128	<0.5	>32
H6	4	8	≥16	32	≥8	>16	2	>128	<0.5	32
H 7	2	16	0.5	32	0.5	8	2	128	8	16
H8	2	128	<0.25	32	<0.12	8	2	128	2	16
H9	2	128	≥16	32	≥8	16	2	16	0.5	4
H10	2	32	≥16	16	0.125	>16	<1	128	<0.5	32

Table S3. A. The percentage mortality of larvae caused by the tested *S. pettenkoferi* strains and the *S. aureus* strain ATCC43300 (used as a positive control) in 120 hours after injection into larvae hemocoel; B. Fifty percent lethal dose (LD50) values of by the tested strains.

A.

**Bacterial strain**	**The number [n] of live larvae after a specified period following injection**					**Percentage mortality after 120h[%]**
	24h	48h	72h	96h	120h	
	Bacterial inoculum =1.0 OD600					
H9	9.7	9.7	9.7	9.7	9.7	3
C1	10	9.7	9.7	9.7	9.7	3
C2	10	9.7	9.7	9.7	9.7	3
H6	10	10	10	9.7	9.7	3
Sa43300	0	0	0	0	0	100
	Bacterial inoculum =0.5 OD600					
H9	9.7	9.7	9.7	9.3	9.3	7
C1	10	9.7	9.3	9.3	9.3	7
C2	9.7	9.7	9.7	9.7	9.7	3
H6	10	9.7	9.7	9.3	8,7	13
Sa43300	7	6	4	3	3	70
	Bacterial inoculum =0.1 OD600					
H9	10	9.3	9.3	9.3	9.3	7
C1	9.3	9.3	9.3	9.3	9.3	7
C2	10	10	10	10	9,7	3
H6	10	10	10	10	10	0
Sa43300	7.7	7.3	7	6.7	6	40
	Bacterial inoculum =0.01 OD600					
H9	10	10	10	10	10	0
C1	9.3	9	9	9	9	10
C2	9.7	9.7	9.7	9.3	9.3	7

H6	9.7	9.7	9.7	9.7	9.7	3
Sa43300	9	9	8.7	8.7	8.3	17

B.

Bacterial strain	50% lethal dose [CFU/Ml]
H9	$1.52x10^{8}$
C1	$2.42x10^{14}$
C2	$3.07x10^{8}$
H6	$4.53x10^{9}$
Sa43300	$1.76x10^{5}$

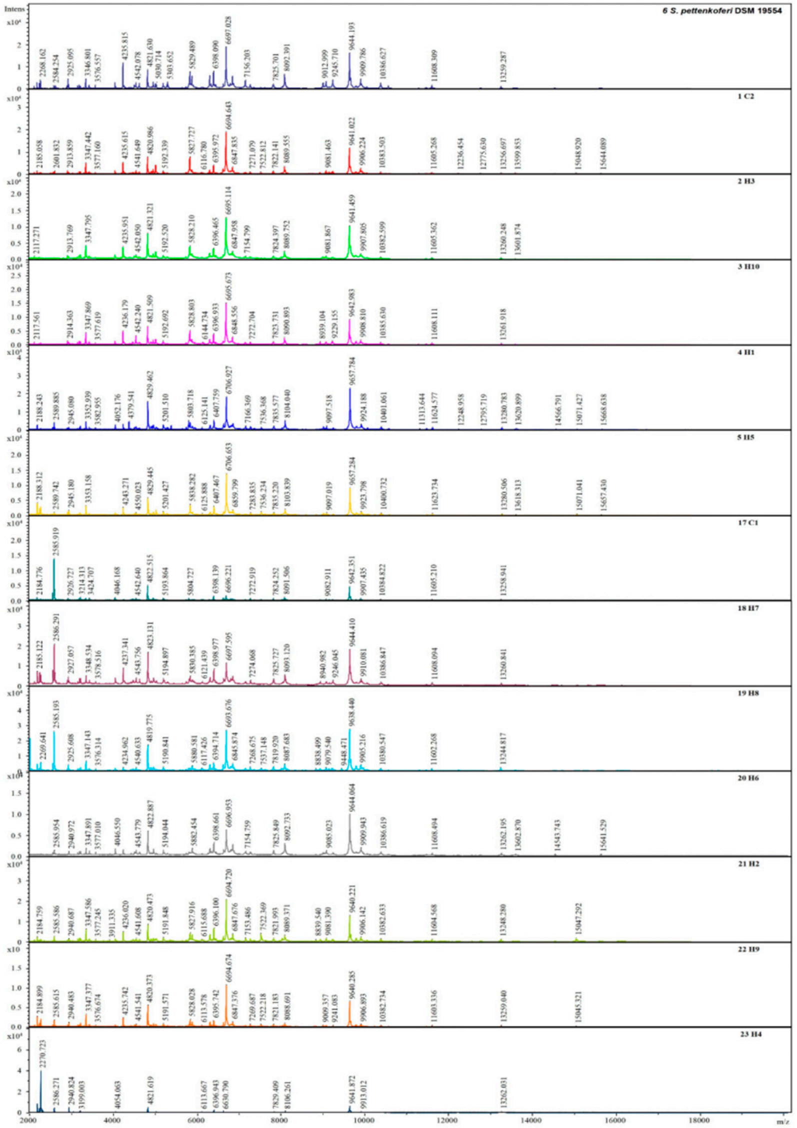

Figure S1. MALDI-TOF spectra comparison of all investigated *S. pettenkoferi* strains.

A)

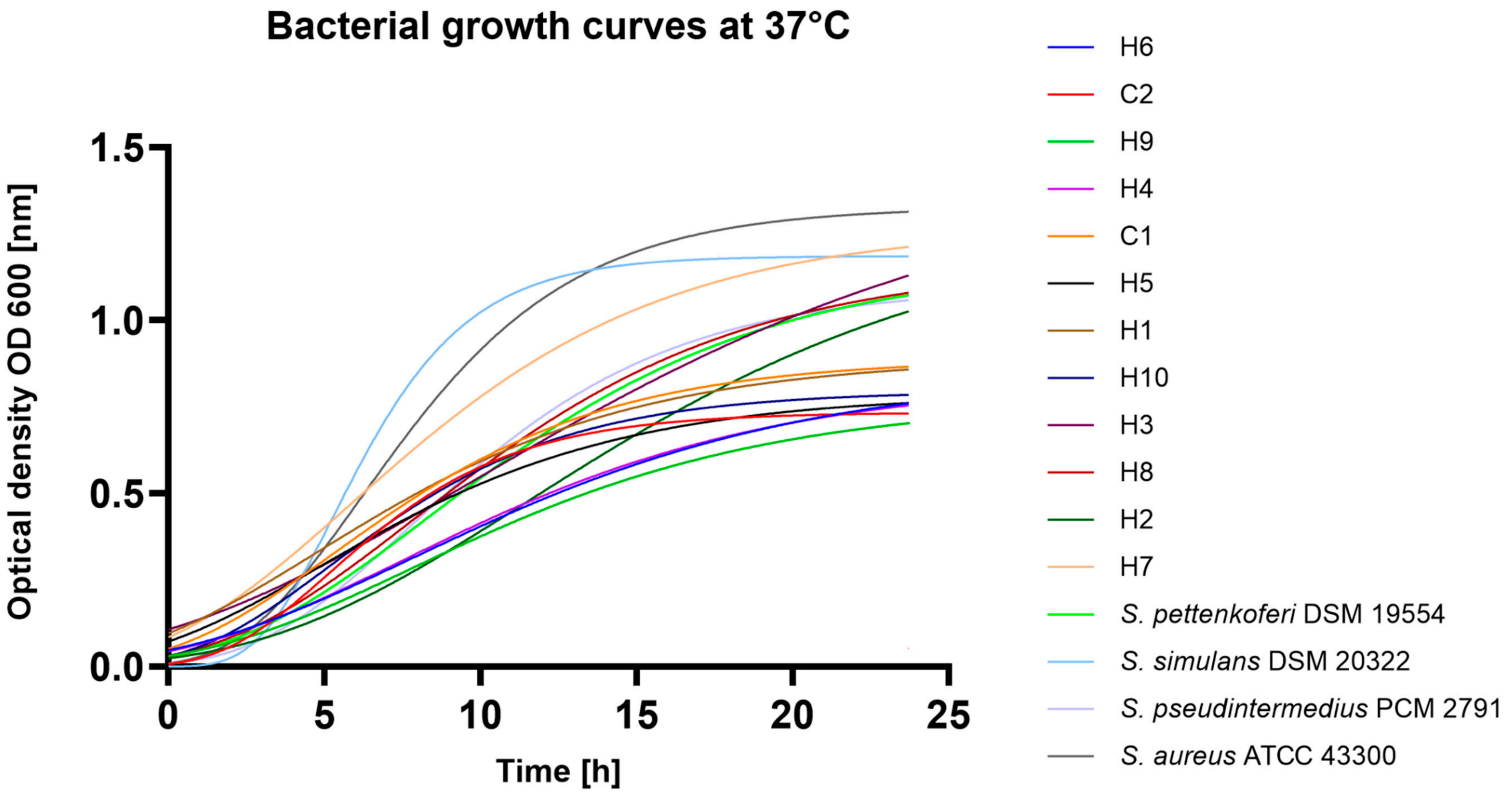
Bacterial growth curves at 37°C
Optical density OD 600 [nm]
1.5
1.0
0.5
0.0
0
5
10
15
20
25
Time [h]
H6
C2
H9
H4
C1
H5
H1
H10
H3
H8
H2
H7
S. pettenkoferi DSM 19554
S. simulans DSM 20322
S. pseudintermedius PCM 2791
S. aureus ATCC 43300

B)

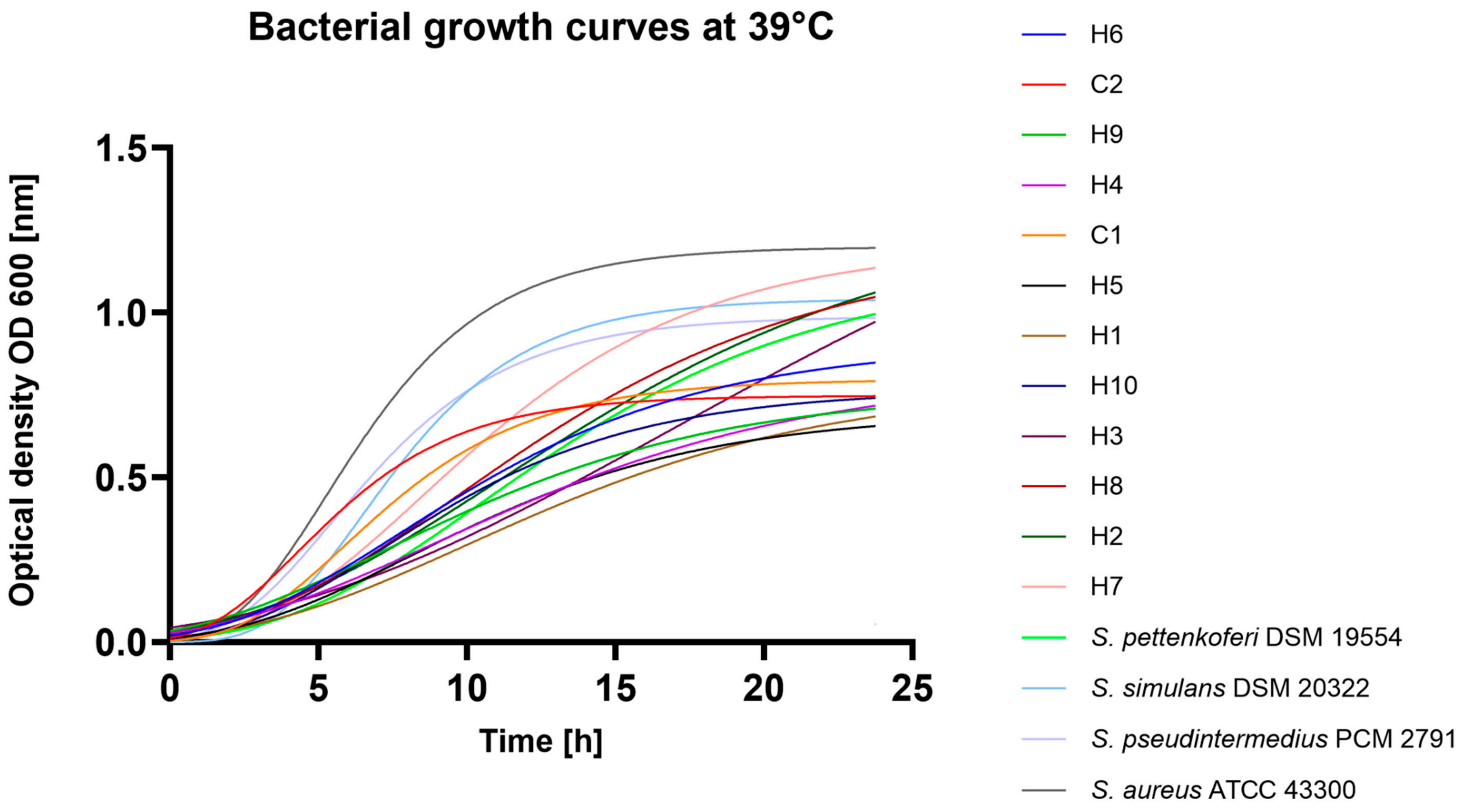

Figure S2. The growth curves for all *S. pettenkoferi* strains under investigation (C1, C2; H1-H10) and chosen reference strains in A) 37°C and B) 39°C.

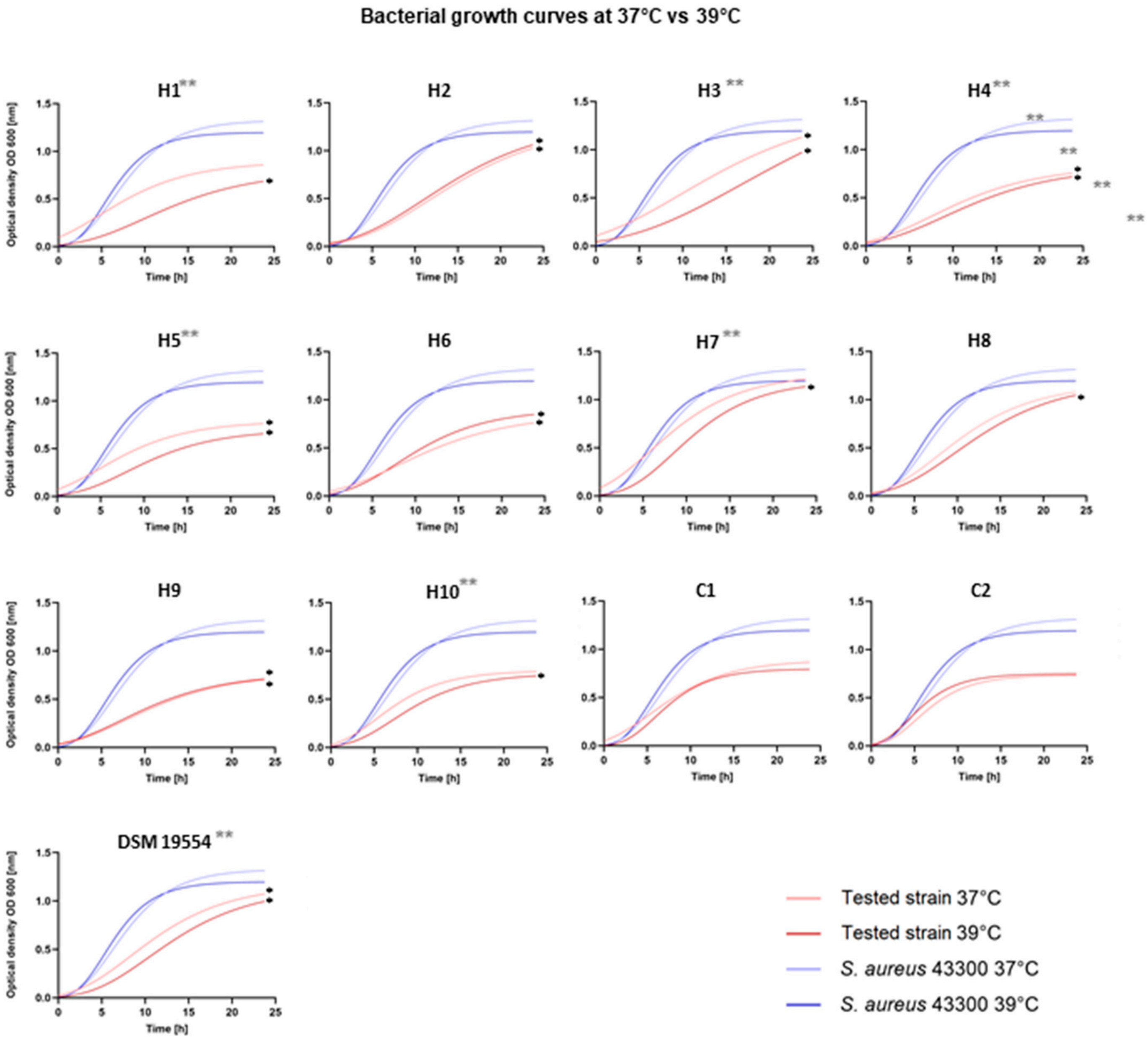

* a significant difference in growth compared to *S. aureus* ATCC 43300; $p<0.05$

** a significant difference in growth in 37℃ and 39℃; $p<0.05$

Figure S3. The growth of all *S. pettenkoferi* in 37℃ and 39℃ in comparison to S. aureus ATCC 43300 in 37℃ and 39℃.
