# Supplementary material for: Comparison of Staphylococcus pettenkoferi Isolated from Human Clinical Cases and Cat Carriers Regarding Antibiotic Susceptibility and Biofilm Production
Source: Int J Mol Sci. 2025 Feb 24;26(5):1948. doi: 10.3390/ijms26051948 (PMC11900592; doi:10.3390/ijms26051948)
Supplement: Supplementary file 1 [file ijms-26-01948-s001.zip › Supplementary Materials Figure S5.pdf]

Figure S5: Growth curves of bacterial strains used in the current study at 39°C over a 24-hour period. Optical density (OD) at 600 nm was measured at hourly intervals. The data represent the mean OD values, with error bars indicating the standard deviation (SD), and the corresponding generation times (GT) ± SD.

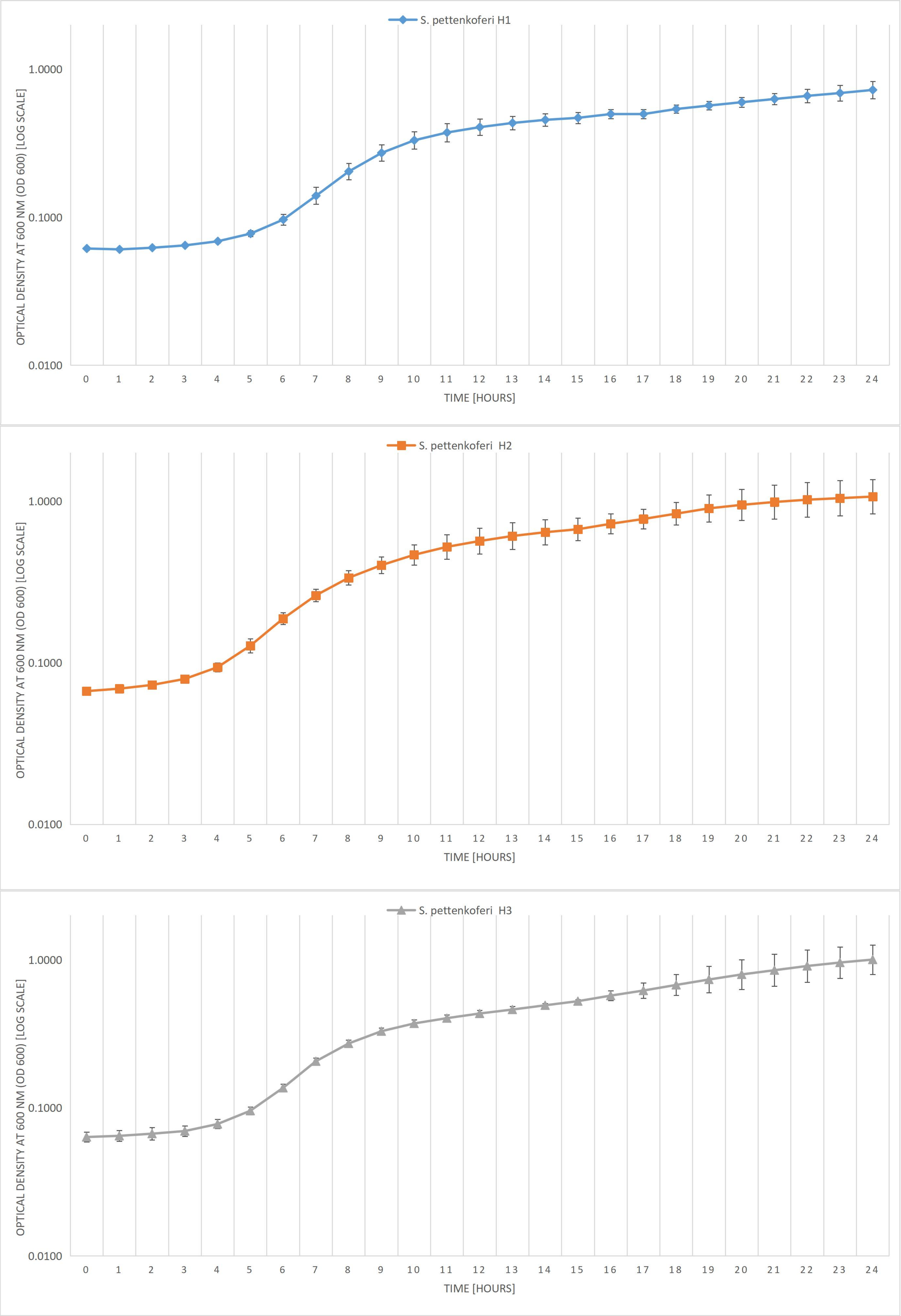

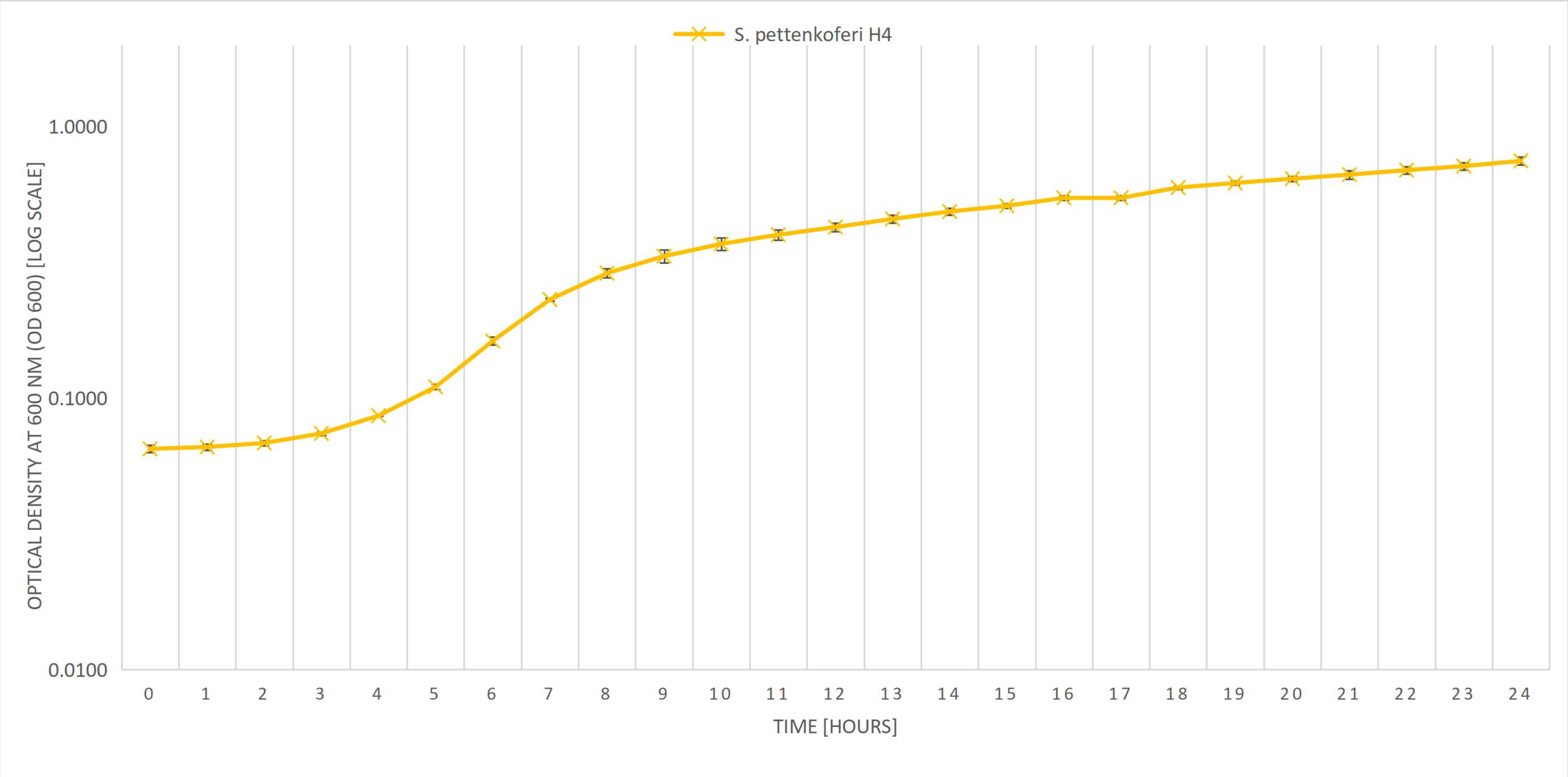
S. pettenkoferi H4
OPTICAL DENSITY AT 600 NM (OD 600) [LOG SCALE]
1.0000
0.1000
0.0100
0 1 2 3 4 5 6 7 8 9 10 11 12 13 14 15 16 17 18 19 20 21 22 23 24
TIME [HOURS]

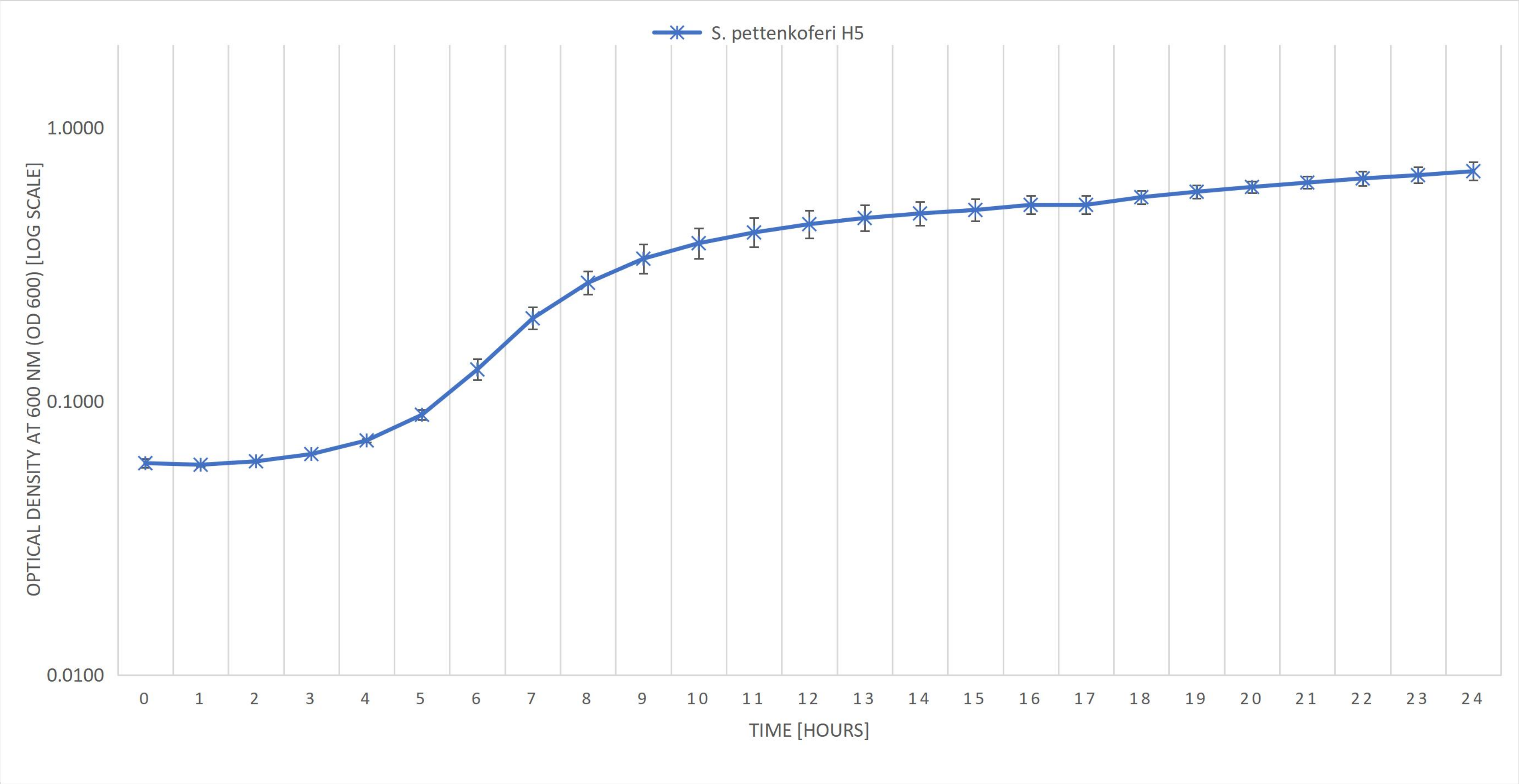
S. pettenkoferi H5
OPTICAL DENSITY AT 600 NM (OD 600) [LOG SCALE]
1.0000
0.1000
0.0100
0 1 2 3 4 5 6 7 8 9 10 11 12 13 14 15 16 17 18 19 20 21 22 23 24
TIME [HOURS]

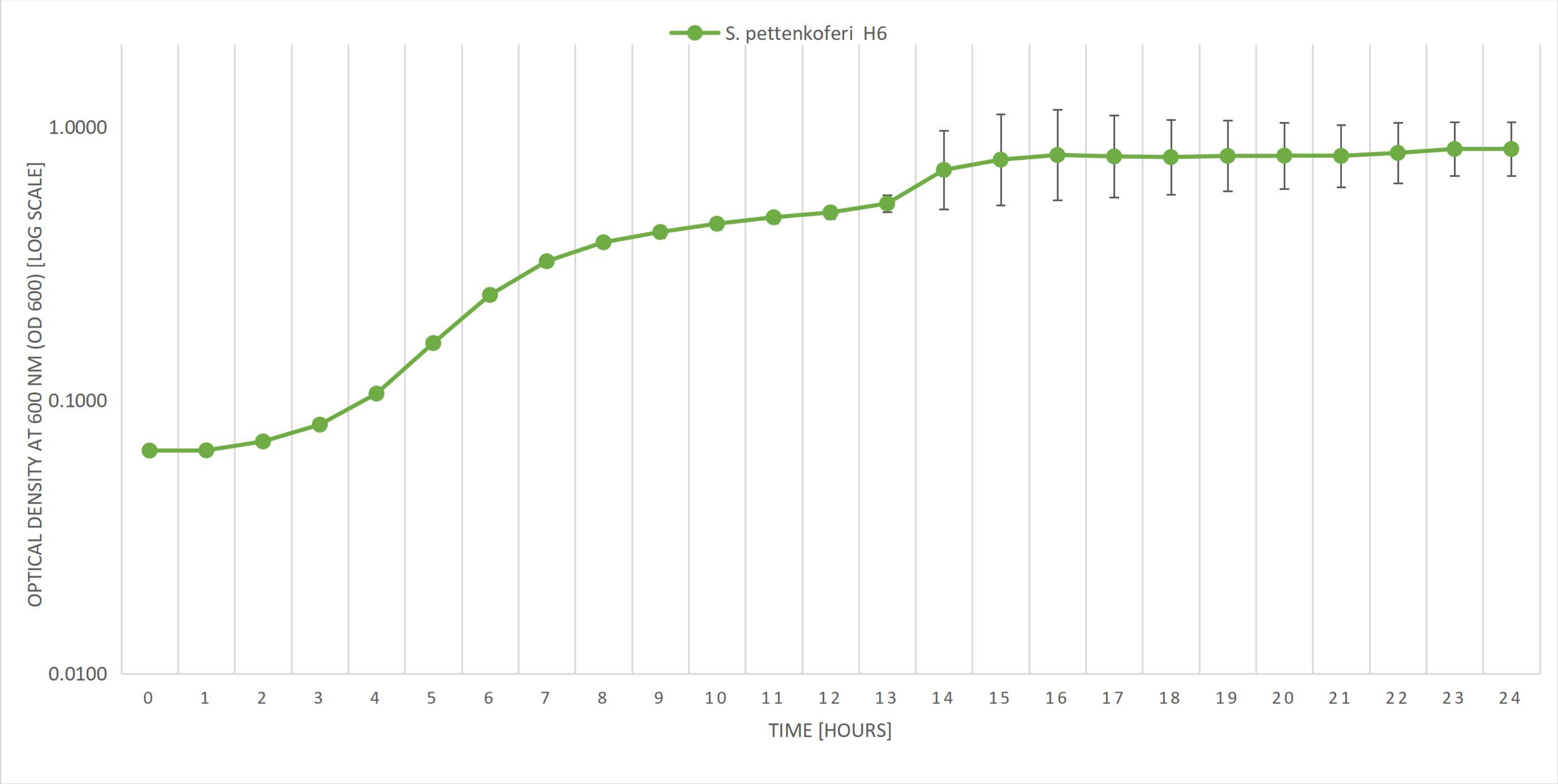
S. pettenkoferi H6
OPTICAL DENSITY AT 600 NM (OD 600) [LOG SCALE]
1.0000
0.1000
0.0100
0 1 2 3 4 5 6 7 8 9 10 11 12 13 14 15 16 17 18 19 20 21 22 23 24
TIME [HOURS]

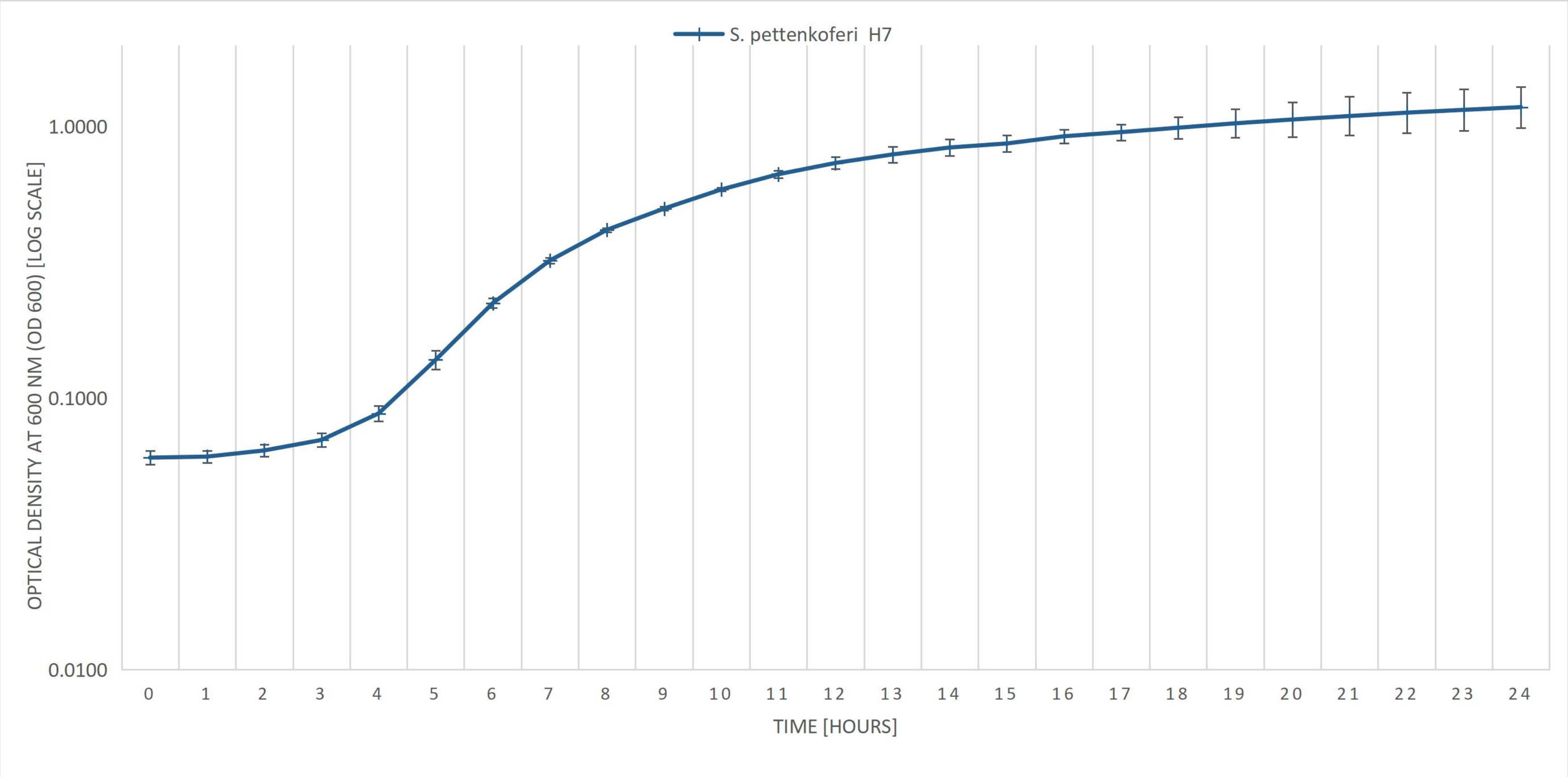
S. pettenkoferi H7
OPTICAL DENSITY AT 600 NM (OD 600) [LOG SCALE]
1.0000
0.1000
0.0100
0 1 2 3 4 5 6 7 8 9 10 11 12 13 14 15 16 17 18 19 20 21 22 23 24
TIME [HOURS]

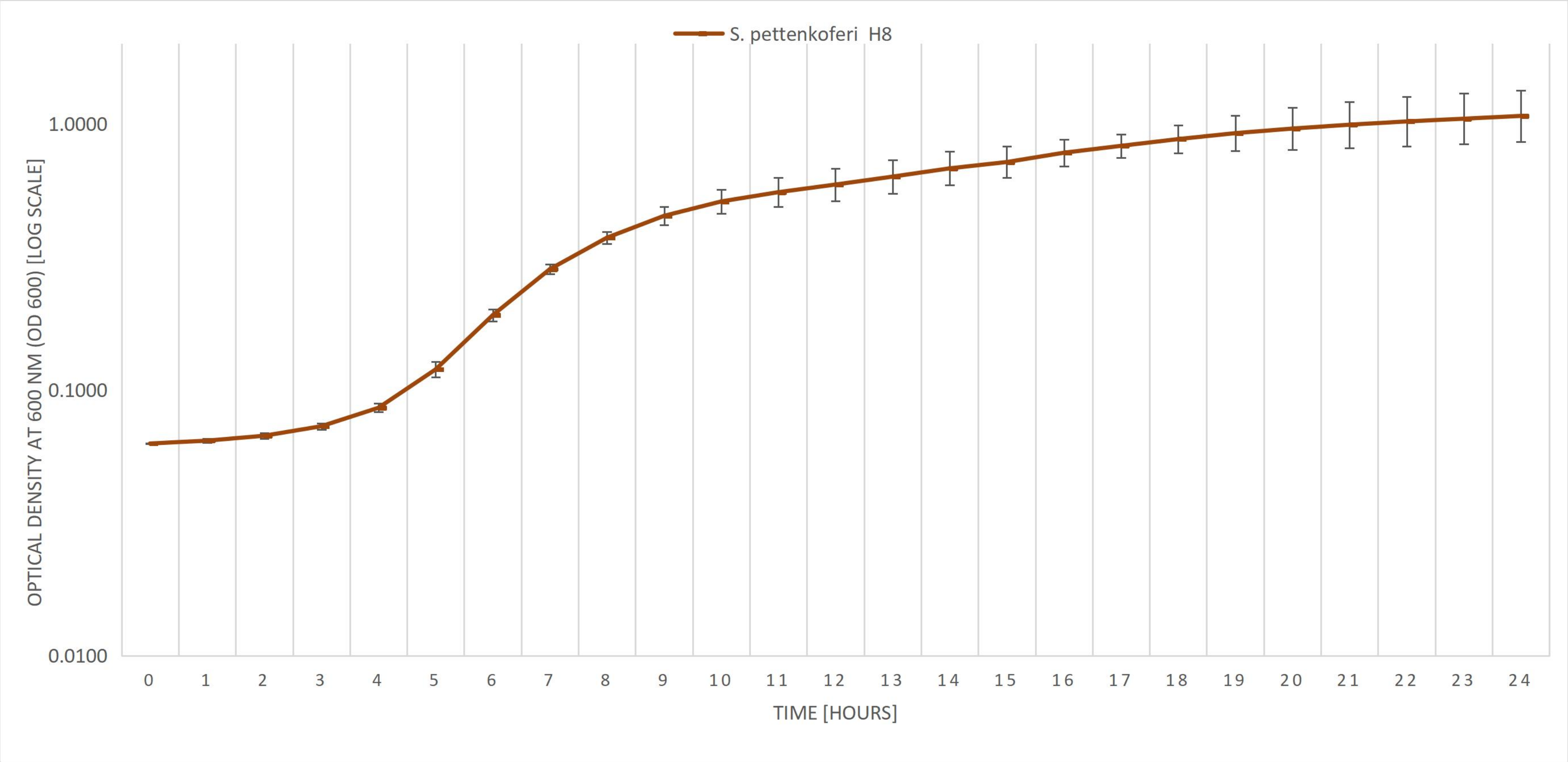
S. pettenkoferi H8
OPTICAL DENSITY AT 600 NM (OD 600) [LOG SCALE]
1.0000
0.1000
0.0100
0 1 2 3 4 5 6 7 8 9 10 11 12 13 14 15 16 17 18 19 20 21 22 23 24
TIME [HOURS]

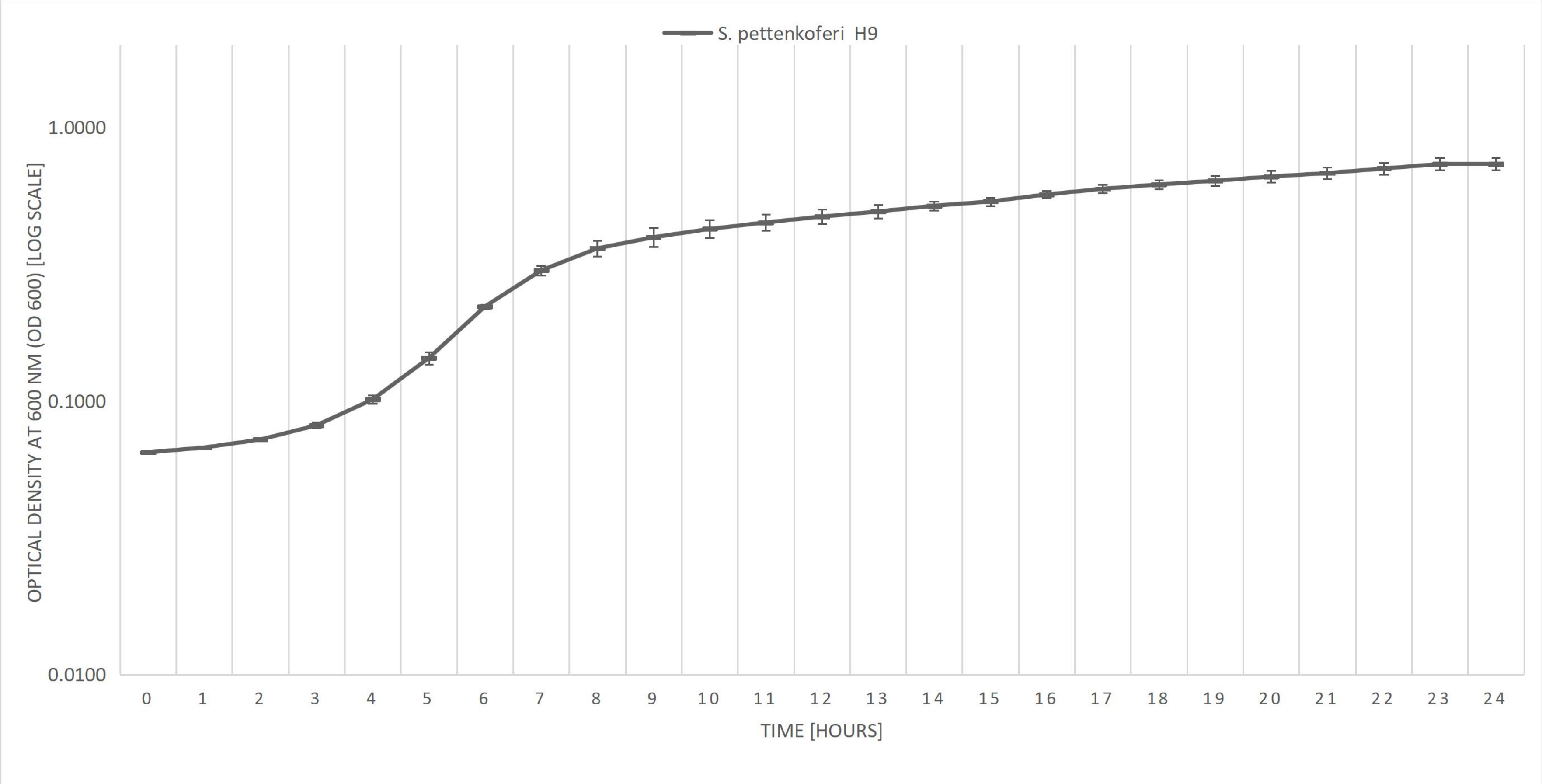
S. pettenkoferi H9
OPTICAL DENSITY AT 600 NM (OD 600) [LOG SCALE]
1.0000
0.1000
0.0100
0 1 2 3 4 5 6 7 8 9 10 11 12 13 14 15 16 17 18 19 20 21 22 23 24
TIME [HOURS]

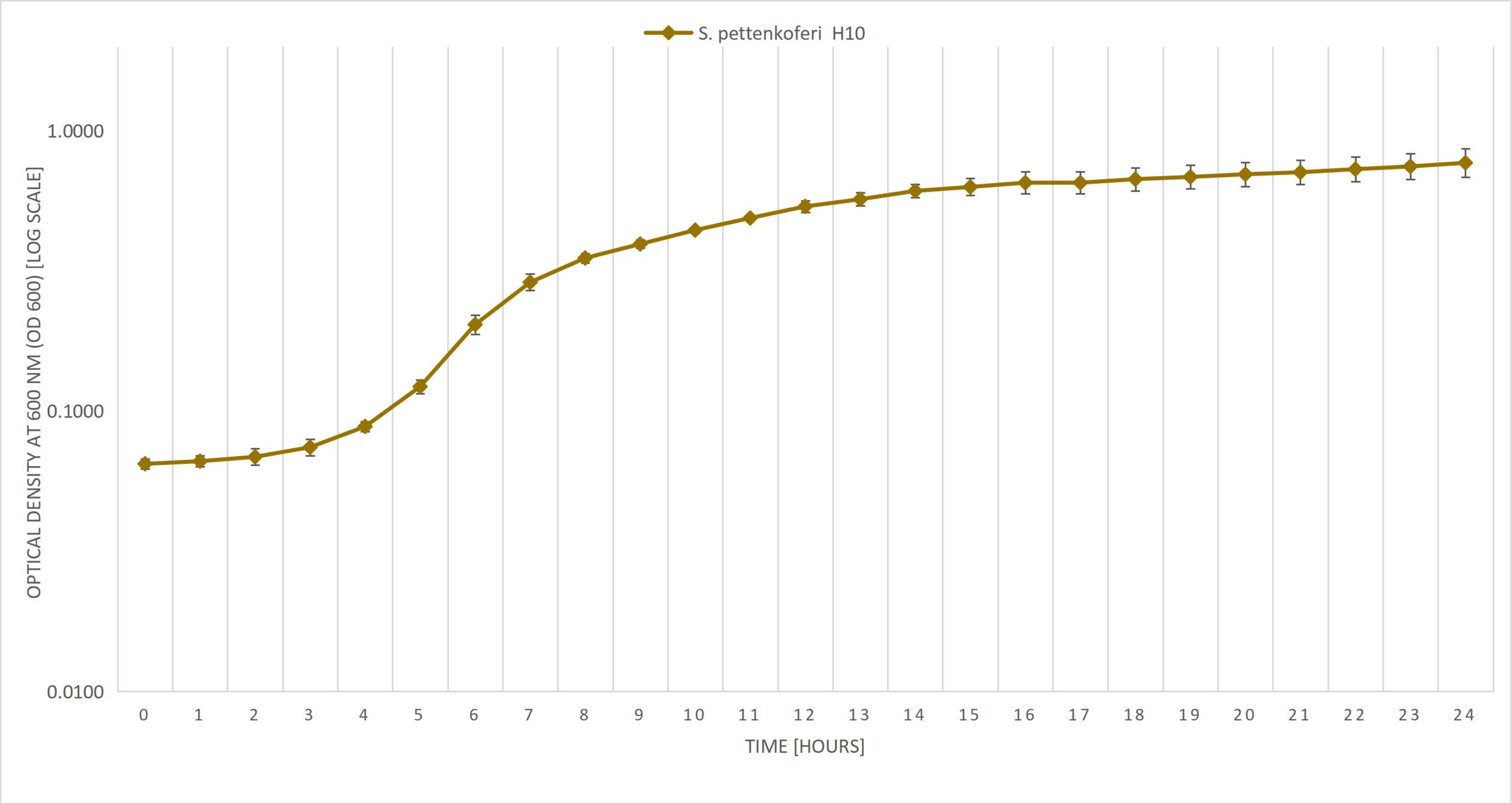
S. pettenkoferi H10
OPTICAL DENSITY AT 600 NM (OD 600) [LOG SCALE]
1.0000
0.1000
0.0100
0 1 2 3 4 5 6 7 8 9 10 11 12 13 14 15 16 17 18 19 20 21 22 23 24
TIME [HOURS]

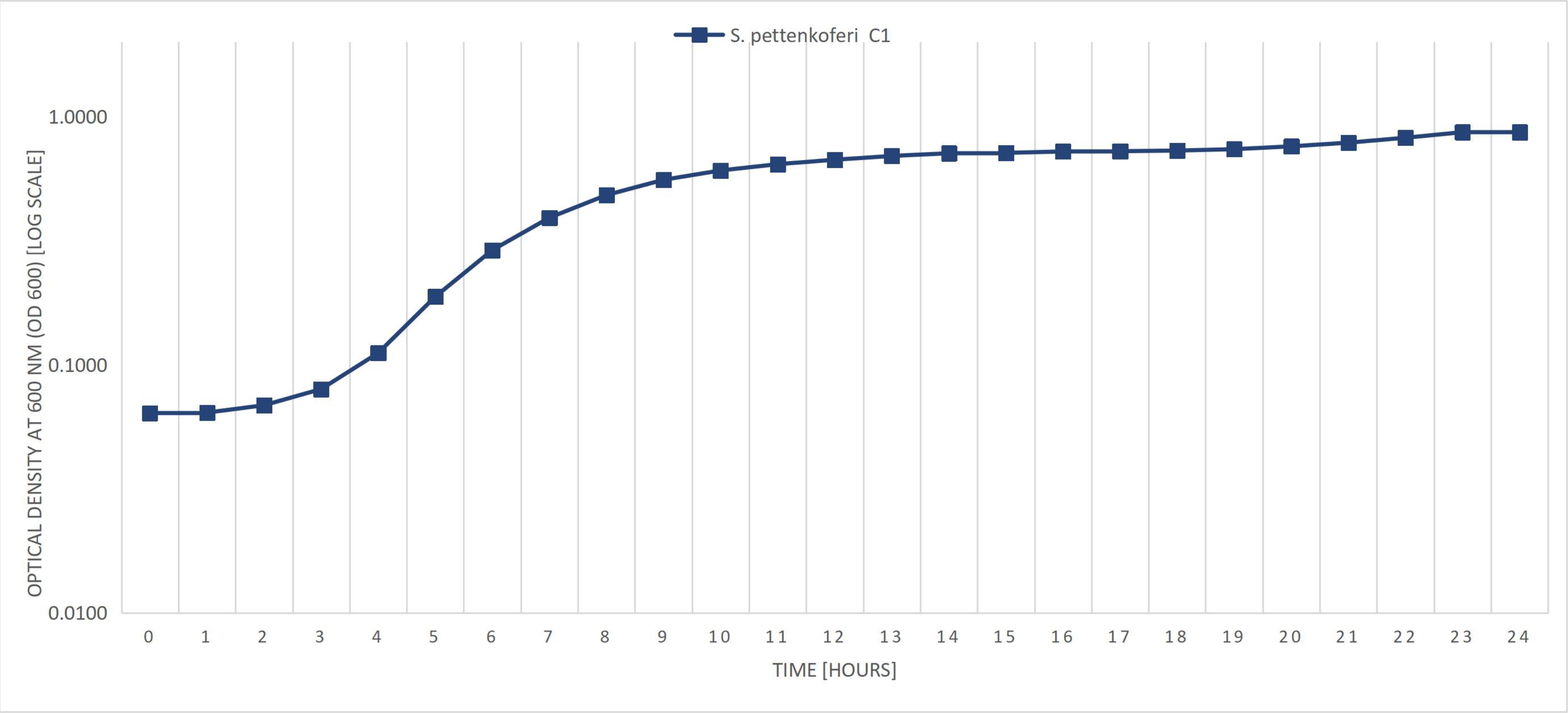
S. pettenkoferi C1
OPTICAL DENSITY AT 600 NM (OD 600) [LOG SCALE]
1.0000
0.1000
0.0100
0 1 2 3 4 5 6 7 8 9 10 11 12 13 14 15 16 17 18 19 20 21 22 23 24
TIME [HOURS]

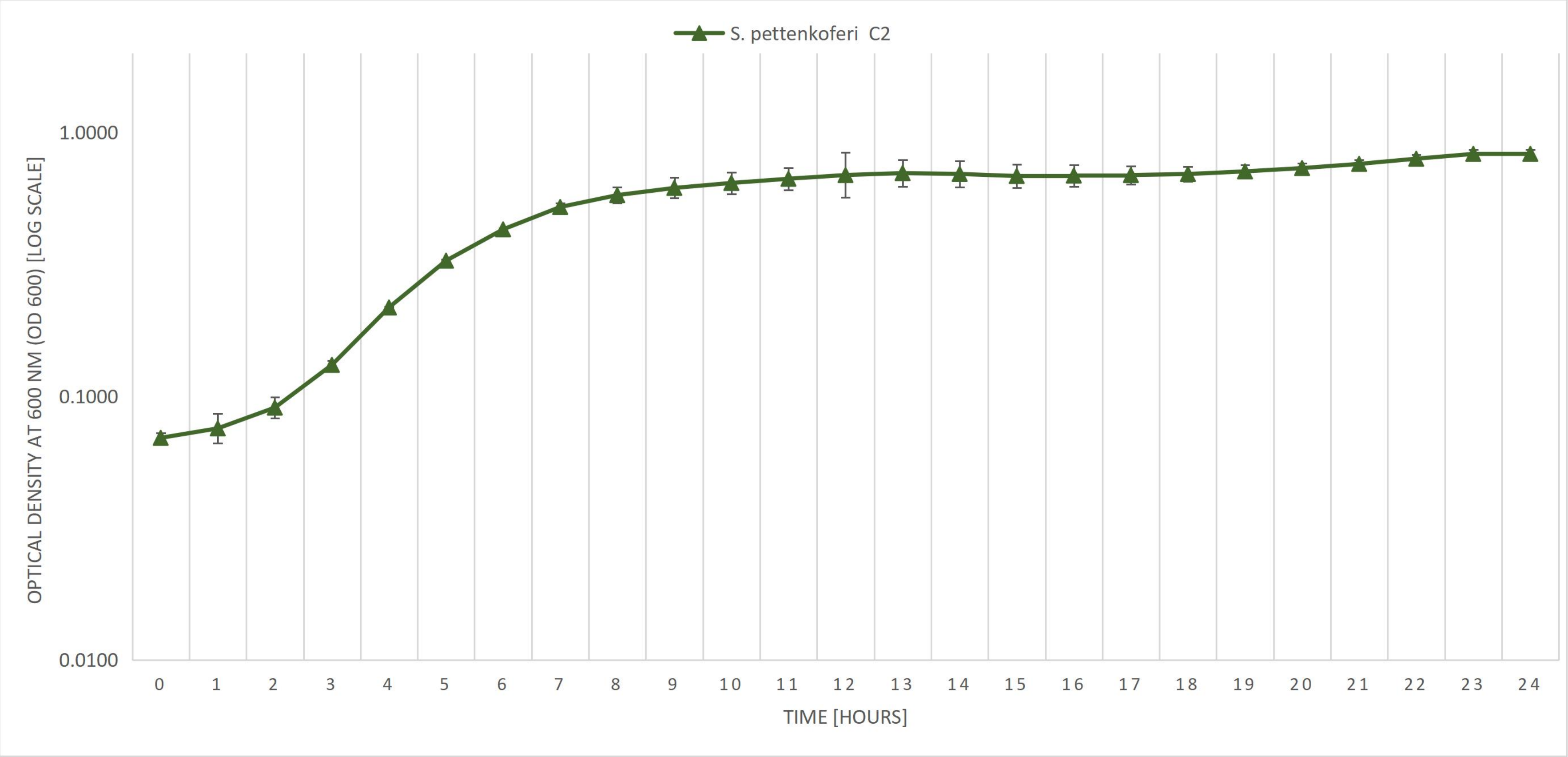
S. pettenkoferi C2
OPTICAL DENSITY AT 600 NM (OD 600) [LOG SCALE]
1.0000
0.1000
0.0100
0 1 2 3 4 5 6 7 8 9 10 11 12 13 14 15 16 17 18 19 20 21 22 23 24
TIME [HOURS]

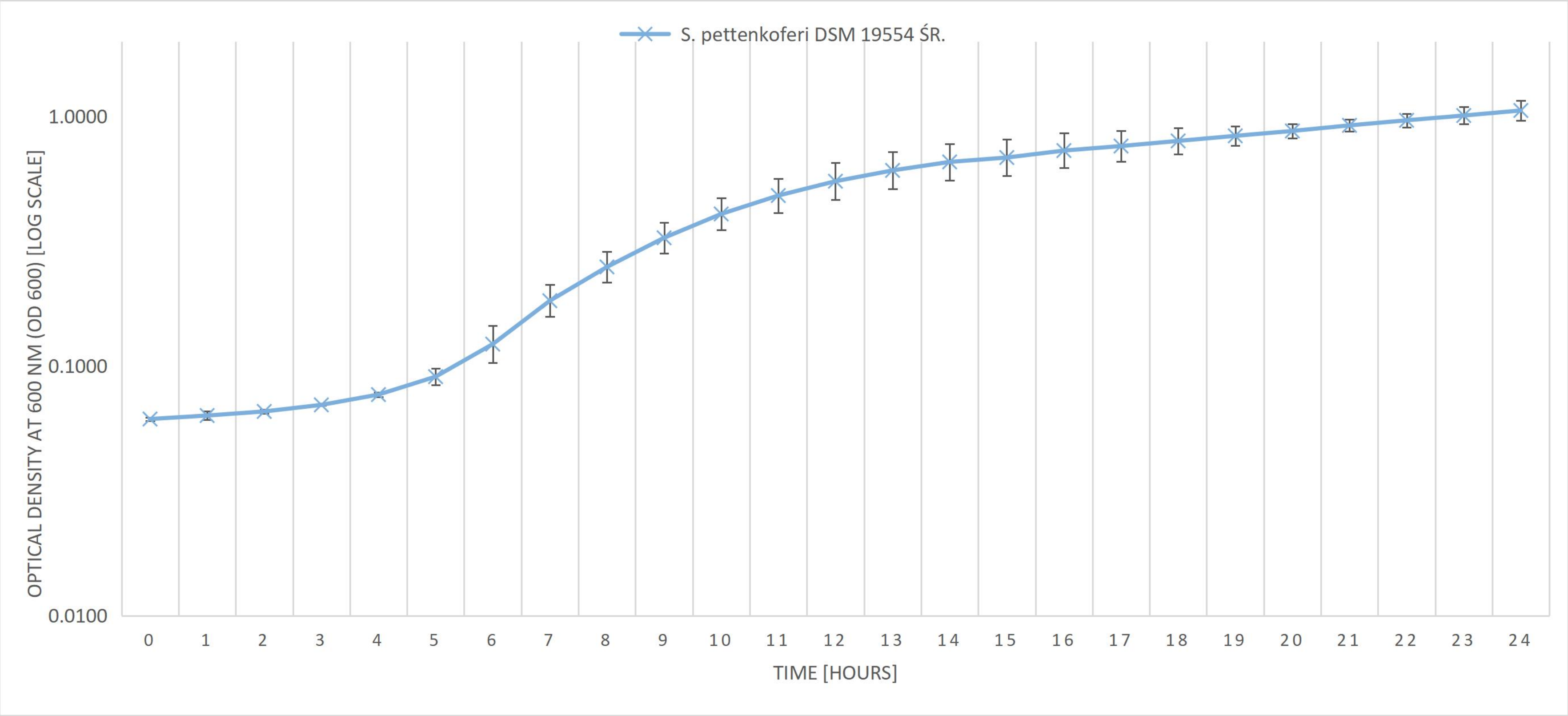
S. pettenkoferi DSM 19554 ŚR.
OPTICAL DENSITY AT 600 NM (OD 600) [LOG SCALE]
1.0000
0.1000
0.0100
0 1 2 3 4 5 6 7 8 9 10 11 12 13 14 15 16 17 18 19 20 21 22 23 24
TIME [HOURS]

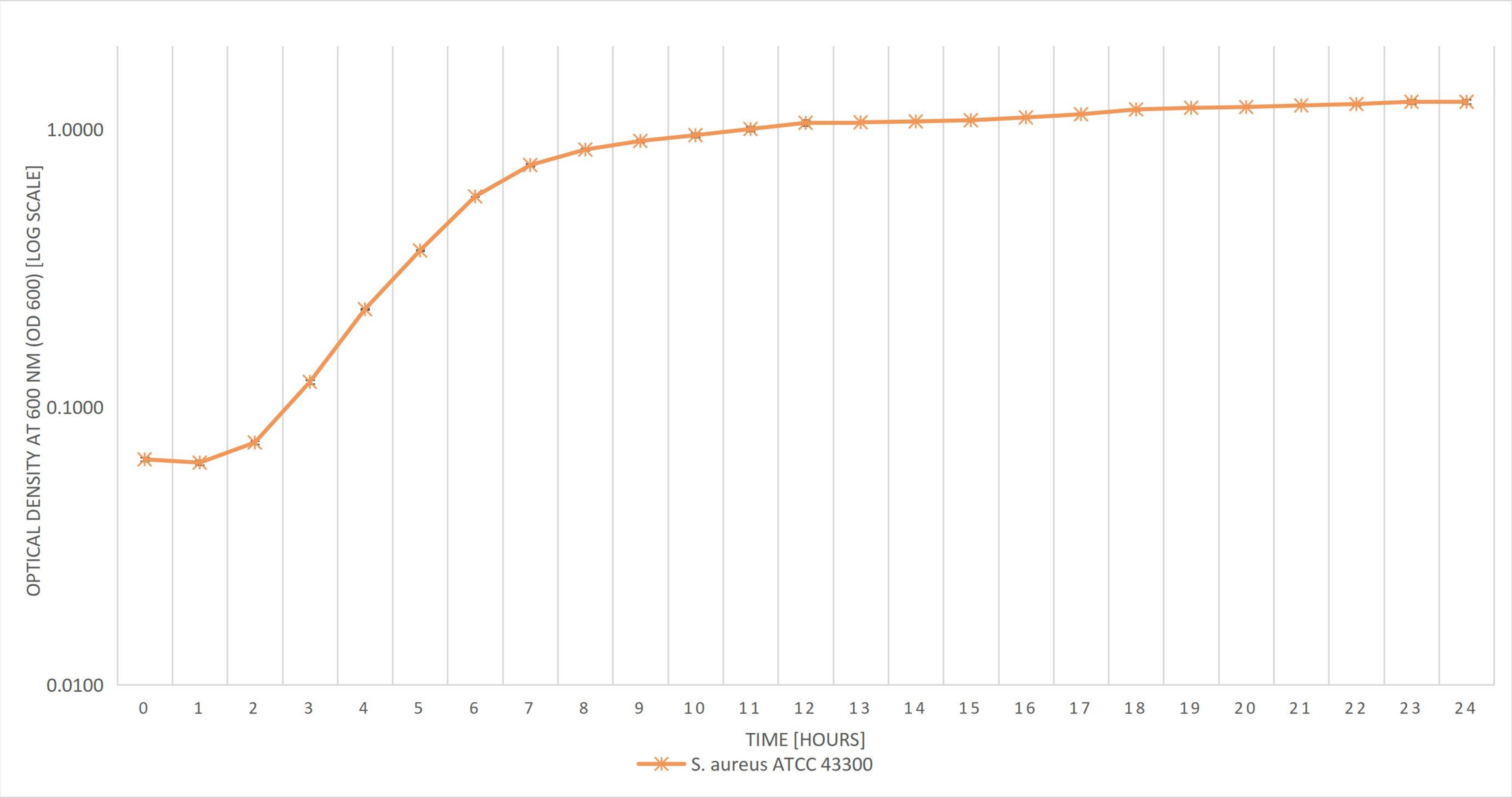
OPTICAL DENSITY AT 600 NM (OD 600) [LOG SCALE]
1.0000
0.1000
0.0100
0 1 2 3 4 5 6 7 8 9 10 11 12 13 14 15 16 17 18 19 20 21 22 23 24
TIME [HOURS]
S. aureus ATCC 43300

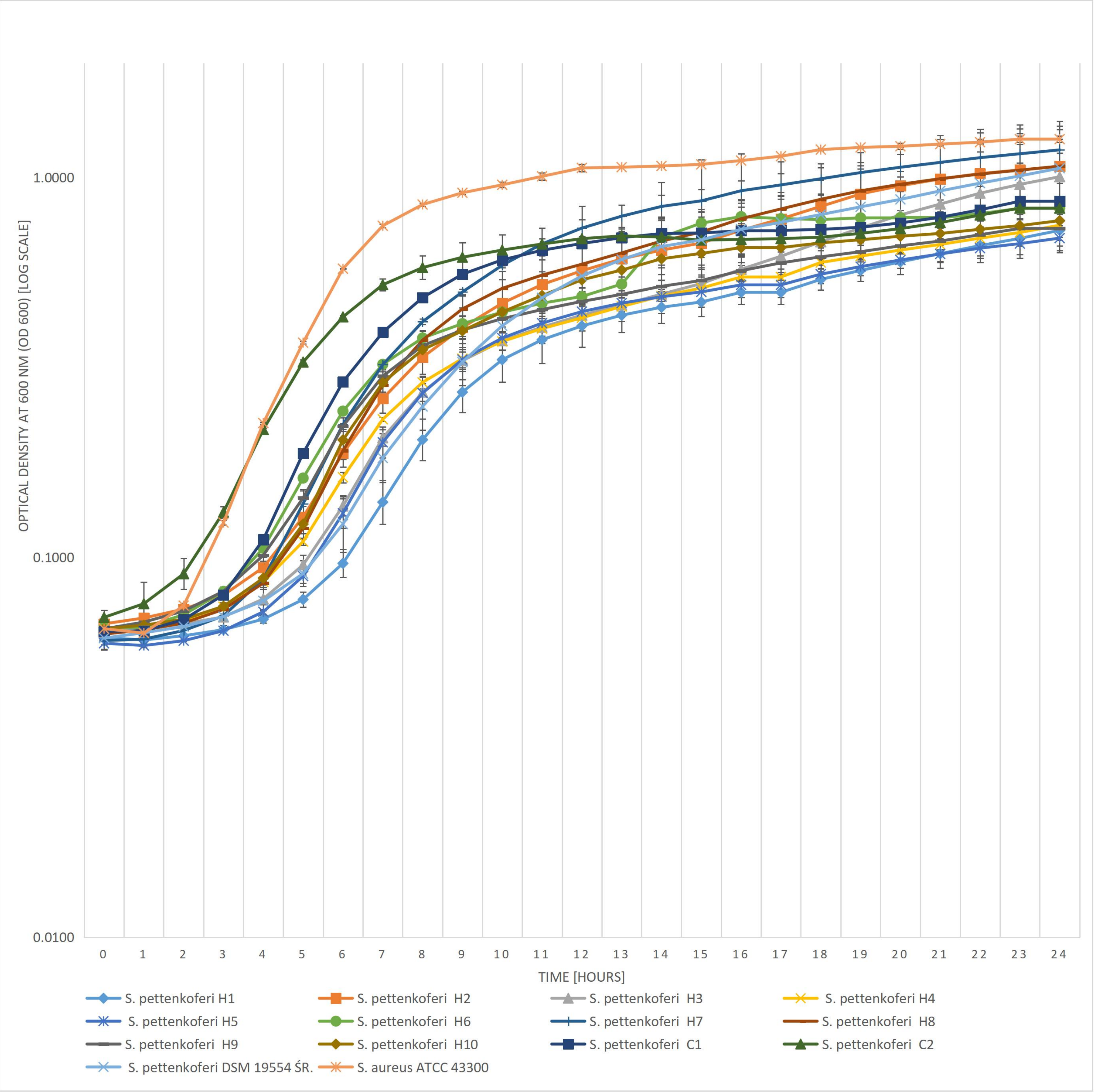
1.0000
0.1000
0.0100
OPTICAL DENSITY AT 600 NM (OD 600) [LOG SCALE]
0 1 2 3 4 5 6 7 8 9 10 11 12 13 14 15 16 17 18 19 20 21 22 23 24
TIME [HOURS]
S. pettenkoferi H1
S. pettenkoferi H2
S. pettenkoferi H3
S. pettenkoferi H4
S. pettenkoferi H5
S. pettenkoferi H6
S. pettenkoferi H7
S. pettenkoferi H8
S. pettenkoferi H9
S. pettenkoferi H10
S. pettenkoferi C1
S. pettenkoferi C2
S. pettenkoferi DSM 19554 ŚR.
S. aureus ATCC 43300
